# The Roadmap of Graphene-Based Sensors: Electrochemical Methods for Bioanalytical Applications

**DOI:** 10.3390/bios12121183

**Published:** 2022-12-19

**Authors:** Ghazala Ashraf, Ayesha Aziz, Tayyaba Iftikhar, Zi-Tao Zhong, Muhammad Asif, Wei Chen

**Affiliations:** 1 Britton Chance Center for Biomedical Photonics at Wuhan National Laboratory for Optoelectronics-Hubei Bioinformatics & Molecular Imaging Key Laboratory, Department of Biomedical Engineering, College of Life Science and Technology, Huazhong University of Science and Technology, Wuhan 430074, China; 2 Key Laboratory of Material Chemistry for Energy Conversion and Storage, Ministry of Education, Hubei Key Laboratory of Material Chemistry and Service Failure, School of Chemistry and Chemical Engineering, Huazhong University of Science and Technology, Wuhan 430074, China; 3 Hubei Key Laboratory of Plasma Chemistry and Advanced Materials, School of Materials Science and Engineering, Wuhan Institute of Technology, Wuhan 430205, China

**Keywords:** bioanalyte, cancer, EC sensor, graphene, immunosensors, live cells, nanomaterials, neurotransmitters

## Abstract

Graphene (GR) has engrossed immense research attention as an emerging carbon material owing to its enthralling electrochemical (EC) and physical properties. Herein, we debate the role of GR-based nanomaterials (NMs) in refining EC sensing performance toward bioanalytes detection. Following the introduction, we briefly discuss the GR fabrication, properties, application as electrode materials, the principle of EC sensing system, and the importance of bioanalytes detection in early disease diagnosis. Along with the brief description of GR-derivatives, simulation, and doping, classification of GR-based EC sensors such as cancer biomarkers, neurotransmitters, DNA sensors, immunosensors, and various other bioanalytes detection is provided. The working mechanism of topical GR-based EC sensors, advantages, and real-time analysis of these along with details of analytical merit of figures for EC sensors are discussed. Last, we have concluded the review by providing some suggestions to overcome the existing downsides of GR-based sensors and future outlook. The advancement of electrochemistry, nanotechnology, and point-of-care (POC) devices could offer the next generation of precise, sensitive, and reliable EC sensors.

## 1. Introduction

**Figure 1:**
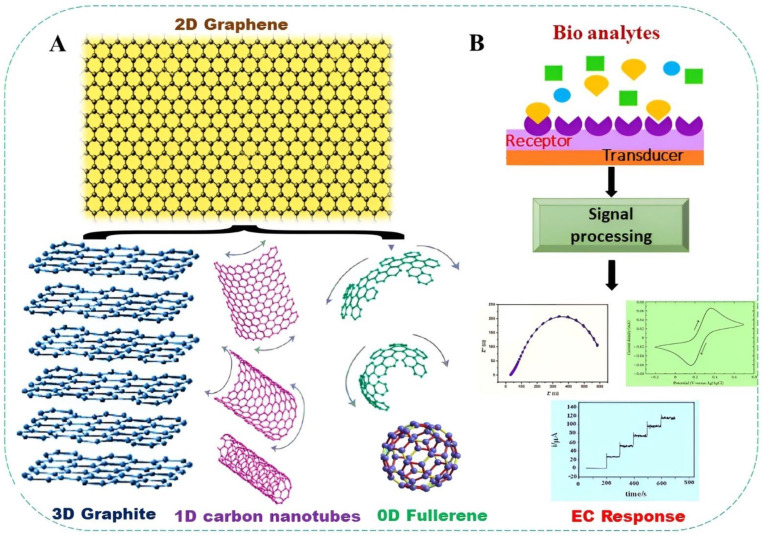
(**A**) 2D GR as the primary constituent of various carbon−based materials. (**B**) A simplistic representation of various components involved in detecting bioanalytes using EC sensor and related transducer responses.

Graphene (GR) is a two-dimensional 2D, single layer of Sp^2^ hybridized carbon atoms meticulously packed in a hexagonal lattice assembly [1]. Owing to this electronic configuration, GR possesses unique properties, including zero bandgaps, a large surface area of 2630 m^2^/g, remarkable optical transparency (97.7%), high elasticity, young Modulus (≈1 TPa) mechanical strength, and high conductivity (3K–5K W m^−1^ K^−1^) [2]. As GR establishes an apparent ambipolar electronic field effect and various types of charge transporters (up to 10^13^ cm^−2^ concentration) can be modified by altering gate electrical energy [3,4]. These charge carriers are also known as massless Dirac fermions naturally well-defined by the Dirac equation and can move to micrometers at ambient temperature without scattering [5]. GR can be processed into various geomorphological nanostructures, including nanosheets (NSs), paper-thin films, nanoribbons (NRs), nanotubes (NTs), paper, fiber, sponges, and foams to attain advanced sensitivity and elasticity [6,7,8,9,10]. The main evidence that stable 2D materials can exist in stable crystalline form comes from GR. It can be viewed as the core element of 0D fullerenes, 1D CNTs, and 3D graphite at once, as shown in Figure 1A. These outstanding physicochemical properties support the GR utilization as a model-building substrate in the fabrication of hybrid NMs [11]. Meanwhile, in 2004, Geim and Novoselov reported the characterization of mechanically scaled GR monolayers that offer exceptional and innovative prospects for eclectic applications in the field of catalysis, photocatalysis, supercapacitors, batteries, fuel cells, EC biosensors, and so on [12].

The practice of GR in the devices mentioned above needs well-preserved inherent electric properties, simple incorporation, and homogeneous distribution into several mediums [13,14]. Different methods have been reported for the production of high-quality GR. Examples include mechanical exfoliation of graphite, chemical vapor deposition on Cu, Ni, and Ru substrates, epitaxial growth on silicon carbide, chemical scaling of graphite crystals via its intercalation complexes, and some synthetic chemical procedures [15]. Among these, chemical methods are more economical and facile for the large-scale production of GR from several precursors, which aids its practical application in diverse fields [16]. These procedures mostly comprise graphite oxidation to graphene oxide (GO) and its reduction to graphene (RGO). Biocompatibility, high conductivity, a sizable surface area, and surface-to-air electron transfer make GR-based NMs exceptional candidates for EC studies and their applications exceptional for detecting a wide variety of bioanalytes [17]. Remarkably, GR has demonstrated the capability of thorough electron transport from biomolecules (proteins and enzymes) at the GR-based electrode interface. These characteristics enable the understanding of kinetic electron transfer, thermodynamic properties of electroactive substances at GR-electrode interfaces, and a new path to EC analysis [18,19]. Before going into detail, little knowledge regarding electrochemistry and EC sensors is needed. In general, electrochemistry is concerned with the oxidoreduction reactions that cause microelectronic oscillations at the interface of the working electrode (WE) in an EC system [20].

A typical EC sensor is a detector that assesses the qualitative behavior of one or more analytes in a system and delivers quantitative information about the target analyte. When an electronic state is reorganized at the electrode interface, a sensor includes a recognition element that enables signal measurement regarding differences in resistance or current [21,22]. The biochemical and chemical reactions are raced up and catalyzed by the sensing material on the electrode surface, producing a signal. Figure 1B depicts a schematic of the various EC sensor parts. Leland C. Clark invented an oxygen sensor for EC sensors in the 1950s, separating two electrodes with a membrane [23]. The environmental, commercial, and medical fields can all benefit from the use of this oxygen sensor. Furthermore, the combination of sophisticated sensors and cutting-edge numerical communication system technology enables smart diagnostic tools. The effective integration of a transducer with a recognition component (usually an EC cell with a counter electrode, a reference electrode, and a WE) is a critical component of an EC sensor (i.e., biological or chemical) [24]. Different techniques based on resistance, potential, and electrical current are used to measure EC signals. Voltammetry, conductometry, impedance spectrometry, coulometry, capacitance, and amperometry (AM) are key EC techniques [25,26]. The EC sensors employed in the above-described techniques frequently operate a WE. In this case, GR stands out among other carbonaceous materials as an exceptional modifier in the development of EC sensors. Pristine GR has been used to make a lot of EC sensors, yet these sensors have some downsides because pristine GR tends to collect in the dispersion medium [27,28]. Numerous GR-based nanocomposites (NCs) have been reported to address this disadvantage. It is possible to create GR-based nanostructures (NS) by incorporating GR into metals, metal oxides, and polymers for the detection of cancer biomarkers, nucleotides, microorganisms, neurotransmitters, heavy metals, etc. [29].

Biomolecules range from small to large molecules (such as metabolites/carbohydrates and protein) and are essential building blocks produced by living organisms. Therefore, the occurrence and suitable concentrations of these biomolecules are essential for the proper functioning of living organisms [30,31,32]. Abnormal fluctuations in the concentration of specific biomolecules may result in the malfunction of living bodies. That is why the precise quantification and tracking of the concentration of these biomolecules are vital for timely diagnosis and treatment and guarantee the wellbeing of living organisms [33]. Precisely, numerous approaches have been developed to detect biomolecules, for example, optical methods, chromatographic analysis, capillary electrophoresis, spectrofluorimetric, spectrophotometry, colorimetry, and EC sensors [34,35].

**Scheme 1:**
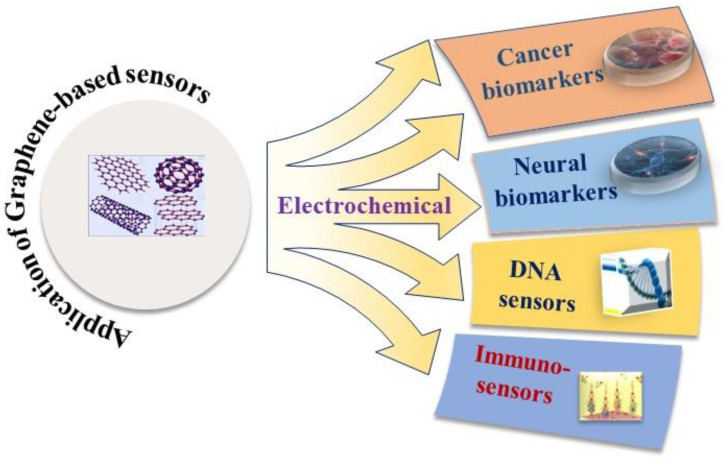
Representation of GR-based diverse range of bioanalytes detection using EC methods discussed in this review.

Among these, EC sensing methods are more worthwhile owing to their compactness, selectivity, sensitivity, and ease of handling compared to the aforementioned techniques [36,37]. Some important biomolecules include pathogens, glucose (Glu), hydrogen peroxide (H_2_O_2_), ascorbic acid (AA), dopamine (DA), uric acid (UA), nitric oxide (NO), hydrogen sulfide (H_2_S), serotonin (ST), acetylcholine (Ach), norepinephrine (NE), proteins, and nucleic acids [38,39]. Additionally, apart from single analyte analysis, there is a need for simultaneous detection of different analytes (such as AA, DA, UA, and ST) with high selectivity [40,41]. The oxidation potentials of these bioanalytes are very close and cannot be parted by traditional bare electrodes because of signals overlapping. Therefore, NMs employed in electrodes should display remarkable EC sensing aptitudes. Electrode materials must be stable and should exhibit decent stability that leads to consistency and replicability. Thus, bare electrodes are improved with innovative NMs that show amazing EC properties in biomolecule analysis. The GR-based NCs with different combinations have emerged as powerful tools to produce highly efficient EC sensors [42]. Reviews published in the last few years related to this topic are up to date. However detailed mechanisms and EC methods for a diverse range of biomarkers detection for early disease diagnosis approach were missing. Each review has especially focused on GR characterization, differentiation of enzymatic and nonenzymatic sensors, or details of fabrication methods for GR and miscellaneous applications for environmental pollutant detection, food, drugs, and gas sensing [43,44]. In this review, we have comprehensively discussed the graphene structure, derivatives, and working principle of EC sensors reported in the last 5 years covering exclusively bioanalytes detection as an early disease diagnostic approach. We debated the GR-based materials as electrodes for EC sensing applications in the field of sensing. Following the detailed understanding of the GR, its derivatives, GR simulation and doping, biomolecules, and EC sensing techniques, we have discussed the detection of the diverse range of bioanalytes. GR-based EC sensors such as cancer biomarkers, neurotransmitters, DNA, immunosensors, and other supplementary bioanalytes are discussed (Scheme 1). The working mechanism of topical GR-based EC sensors, advantages, and real-time analysis of these along details to analytical merit of figures for EC sensors are discussed. Next, the review is summarized by providing the comparison, advantages, and future outlook of the field.

## 2. GR and Its Derivatives

### 2.1. Pristine GR

Briefly, GR is a 2D sheet of hexagonal carbon with an atomic layer thickness. Despite the fact that GR is essentially composed of carbon, its edge and basal plane exhibit a variety of EC characteristics. GR edge has better electron mobility rate, specific capacitance, and catalytic power than GR basal plane. To depict pure GR, one, two, or a few layers of GR can be fabricated [6]. A material somewhat having these characteristics can be classified as a GR derivative. Different types of GR derivatives have been fabricated to increase yield and stability and lower the production cost [10]. As mentioned previously, GR is modified with its derivatives and other materials to increase EC performance. Therefore, it is necessary to know the physicochemical properties, structure, and mode of fabrication of these derivatives, which are as follows:

#### 2.1.1. Graphene Oxide (GO)

GO is single-layered material prepared by graphite oxidation, which is inexpensive and abundant. GO is the oxidized form of GR lace-up with oxygen (O_2_) functional groups. It is easy to use since it is soluble in organic and inorganic solvents [45]. It reveals improved selectivity to positive or fractional positive charge transporters when employed in EC sensors. GO is not a high conductor, but there are many procedures (Hummers, Staudenmaier, Brodie, and Hofmann) available to enhance its conductivity. The efficiency of an oxidation method is normally estimated by the C/O_2_ ratio in GO. Simultaneously, the O_2_ groups produce defects in GO that delay the surface-to-air electron passage and confine electrons, leading to insufficient conductivity and high resistance (≈10^12^ Ω/sq). This drawback makes GO less suitable for electrode modification analogous to other GR derivatives. This drawback is frequently resolved using other metals NCs or NPs such as Cu, Fe, Au, Pt, Mn, Ce, Ti, Co, etc. [46,47].

#### 2.1.2. Reduced Graphene Oxide (RGO)

The reduction of GO obtains RGO. This reduction involves various approaches that can be chemical (hydrazine, AA, Glu, gelatin) reducing agents, EC, thermal, and photoreduction. The ultimate goal is to reduce defects and O_2_ groups in GO and enhance its conductivity [48,49]. Mostly, RGO-based electrode materials in EC sensors show superlative properties compared to GR and GO since it holds high conductivity of GR and negative GO charges [50]. The RGO is well thought out as a possible supporting material for advancing nanohybrid (NH) frameworks owing to its admirable optical and electrical properties.

#### 2.1.3. Graphene Quantum Dots (GQD)

**Figure 2:**
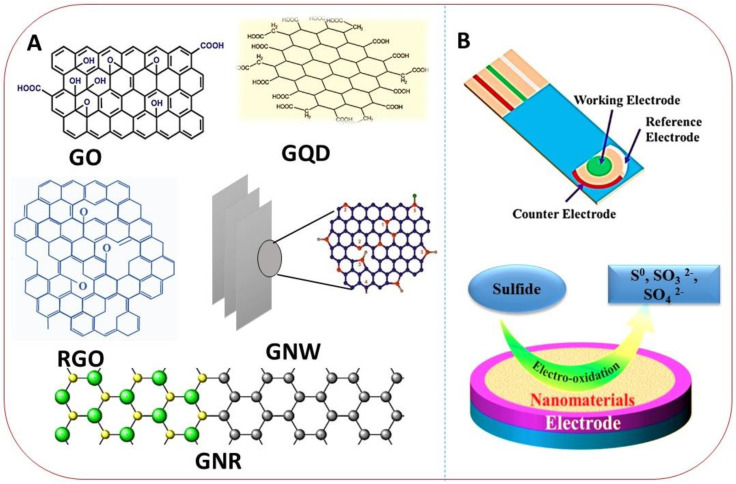
(**A**) Structural demonstration of various GR derivatives. (**B**) A simple depiction of an SPCE and mechanism of EC sulfide oxidation on the electrode surface. Reprinted with permission from Ref. [54].

GQD is an atomic-layered thin GR sheet that possesses (≤20 nm) diameter, high surface area, and improved surface splicing via π–π coupling. In GQD, the electron transfer rate in three dimensions is confined via the quantum confinement effect [51]. Typically, GQD holds carboxylic acid groups at the peripheral parallel GR, hence conveying them with good water solubility and suitability for subsequent functionalization with many inorganic, polymeric, and organic species [52,53]. Owing to the simple structure, health apprehensions, and biotic threats of QDs, the GQDs are the epicenter of substantial efforts to develop low-toxic, biodegradable substitutes with desirable performance in sensing fields. The structural representation of various GR derivatives is displayed in Figure 2A.

#### 2.1.4. Graphene Nanoribbons (GNR)

GNRs are plane, finite, quasi-one-dimensional GR structures. The assembly of their extended edges usually characterizes them: zigzag, armchair, or a transitional character. This control structure has reflective inferences for the electronic properties of GNR. Normally, GNR is semiconductive, but the 1D structures and the bandgap size depend on the edge geometry [55]. The physical, chemical, and structural properties of GNR differ significantly owing to the synthesis approach. Currently, three main methods for GNR fabrication are (a) bottom-up fabrication from polycyclic fragments, (b) cutting from GR by lithography, and (c) unfolding of CNTs [56].

#### 2.1.5. Graphene Nanowalls (GNW)

GNWs can be described as self-organized, sharp-edged, steeply standing, few-layered GO or GR nanosheets are known as carbon nanowalls, carbon nanoflakes, and carbon nanosheets [57]. GNW possesses high stability, surface area, abundantly exposed GR edges, better electric properties, fast electron transport, and high conduction. With these exceptional properties, GNW provides abundant binding positions for target bioanalytes and hence improves the sensitivity of EC sensors [58]. Owing to eminent physical and EC properties along with well-established fabrication approaches, GR and its derivatives have been extensively applied in electrocatalysis, especially in EC sensors. In the next section, the application of GR-based electrode materials and their EC properties for different bioanalytes detection will be discussed.

### 2.2. GR Simulation

Computer-based simulation practices are helpful for learning theoretical tools prior to performing an experiment. These theoretical methods still require a significant amount of computing. Therefore, more effective techniques are required to perform research on ever larger systems. The GR bandgap has received a lot of interest due to its important physicochemical characteristics, which demonstrate how different it is from other allotropic forms of carbon. The 1,3,5-interconnected benzene molecules that form heterocyclic GR configuration were examined using DFT. The EC interactions of GR could point to future modifications in structural configuration [59]. The charged molecules and the interacting carbon surface produce an electron transfer connection, which is reflected in alterations in structural configuration. In simulation methods, individual EC transducer elements can be contained. In order to examine the electrical and atomic configuration of cyclooctatetraene (COT) on GR, first-principles calculations utilizing density functional theory (DFT) were conducted. The GR sheet and COT molecules interact, and conformational fluctuations including a doping hole and a flattened atomic structure have been observed [60]. Due to the shape effect, the dispersion barrier declines as the structural configuration of the GR sheet fluctuates. Similar to benzene, COT has nonplanar symmetric irregular single and double bonds. When COT is charged, a GR field effect transistor may read out the mechanical response, which aids in the creation of a single-molecule EC transducer element. COT molecules get flattened and endowed with a positive charge by altering the GR carrier density. Functional elements showed a decline in energy inversion and band shift, which is the key indicator for sensing the conformational outcome. Though, it transmutes to a two-dimensional shape when reaching negative electrical voltage [61]. Free-standing particles in ionic states were used to investigate the electrical assembly of a COT molecule. Three configurations were discovered: a nonplanar tube-shaped, a planar shape with substitute double and single bonds, and a planar configuration with equal length C-C bonds. These transitions can be employed to forecast how the GR sheet would EC interact with foreign molecules [62].

### 2.3. Graphene Doping

To broaden the spectrum of applications for GR, which is believed to be a material with a zero bandgap, the carrier density must be changed. Numerous doping approaches, including molecule absorption, atom substitution, and self-assembled monolayer deposition, have been shown to be efficient for this purpose. Covalent functionalization of metal NPs, higher electrons/hole concentrations in GR are caused by doping impurities with electron-withdrawing/donating characteristics [63]. The primary technique for developing an adequate band gap in GR is to reduce lattice symmetry. There are three practices to dropping symmetry: chemical doping of the carbon lattice, converting it to GO, and reducing the adjacent dimensions of GR by transforming it into GQDs [64]. According to DFT calculations, doping would dramatically enhance the interaction of GR with a number of gas molecules, including NO, CO, and NO_2_. Niu et al. first demonstrated that codoping nitrogen and silicon on GR networks increases the sensitivity to NO_2_ sensing. In this study, codoped GR NS was fabricated by heating GO composites and an ionic liquid that contained silicon and nitrogen atoms at high temperatures, followed by annealing. Codoping produced GR with p-type features and has a larger sensing response to NO_2_ than thermally reduced GO [65]. A different strategy was used by Zanjani et al. [66], who showed that molecular doping may be used to functionalize GR for more sensitively detecting NH_3_. Although GR molecular doping has outstanding sensing capability, it fails to meet the stability standard and is therefore not practical. Therefore, further modification of GR surface sites must be considered for the practical use of doped GR in sensors in order to overcome the obstacles to chemical doping of GR for devices [67].

## 3. GR-Based EC Sensors for Bioanalytes Detection

### 3.1. Cancer Biomarkers

#### 3.1.1. H_2_S Detection

Endogenous H_2_S is a third gas transmitter indicator produced by enzymatic catalysis of Cys or from sulfide reservoirs in the living system. Several studies have reported that the irregular concentrations of endogenic H_2_S may be associated with diseases, like neurogenic, digestive disorders, breast cancers, and many others [68]. More examination has also been needed on the medical efficacy of H_2_S as a serum biomarker of cardiac diseases. Given the extent of its action, the capability to precisely detect and calculate H_2_S in the biotic environment is vital to further consider the functioning and pharmacology of this prevalent molecule [47,69]. Hence, it is crucial to develop a method for H_2_S detection via consistent, fast, precise, and effective techniques. The simple and economical fabrication of EC sensors allows sensitive and in situ detection of bioanalytes without needing exogenic substances [70]. Ion-sensitive electrodes can quantify sulfide ions (S^2−^) in serum with high selectivity and low DLs (≈0.1 µM). Nevertheless, samples must undergo a basic medium to move proton equilibria from H_2_S (pKa1 = 6.6) toward S^2−^ (pKa2 = 13.8) acid-labile sulfide yielding concentrations typical of the overall sulfide content as an alternative to freely existing H_2_S. On the other hand, reliable AM detection of H_2_S and (HS^−^) is feasible by two-electron oxidation with simple sulfur as a derivative (Equations (1) and (2)) correspondingly [71,72].

H_2_S → S^0^ + 2e^−^ + 2H^+^ (1)

HS^−^ → S^0^ + 2e^−^ + H^+^ (2)

Notably, the development of a protecting sulfur film passivates electrode surface, triggering sensitivity drop and performance inconsistency. Various AM sensors have struggled to resolve this issue by integrating a porous gas membrane and basic internal solution with a redox moderator (i.e., ferrocyanide) which accepts electrons from H_2_S/HS^−^ and is restored at the electrode interface to generate a quantifiable current response (down to 10 nM) [73]. Adel’s group [74] developed a chemiresistive sensor-based on GR attached with a nonconducting polymer, polymethyl methacrylate for H_2_S sensing in a liquid phase. The sensor can unswervingly detect H_2_S in solution without evaporation. The EC performance of the sensor was inspected by EIS, inclusive of high to low frequencies, and promising outcomes in distinguishing several concentrations of H_2_S were noted. Likewise, to illustrate the sensor design, a simple, quaternion elements equivalent circuit with high precision was adopted. Due to the nonexistence of evaporation arrangement, the offered sensing podium was affordable, energy-efficient, compact, and can be cast off in severe environments, e.g., drain systems. Likewise, a poly 4-styrene sulfonic acid (PSS) doped PANI/GR NCs was reported for H_2_S detection. The PSS was prepared at −50 °C and used as a dopant and binding agent between PANI/GR sheets in (a water–chloroform) system. The characterization of fabricated NCs by XRD, Raman, and XPS displayed the π–π stacking flanked by PANI quinoid rings, and the basal planes of GR were strengthened due to PSS existence. The CV, EIS, and AM records showed that the PSS/PANI/GR modified sensor owned charge conversion connections that were more robust than other control NCs without PSS. The solution-based, flexible sensor exhibited high sensitivity to H_2_S with a low 1 pM, a quick retrieval time (150 s), a fast response to several H_2_S concentrations, and high interference aptitude toward other analytes [75].

Electrode fouling and interfering species from biological mediums are the foremost challenges in advancing in situ H_2_S devices. To avoid fouling, a robust electrode constructed on (RGO-MoS_2_) and *o*-phenylenediamine was developed. The modified electrode catalyzed H_2_S oxidation at a lower overpotential (+0.15 V), [76]. The RGO-MoS_2_ improved the EC signal and aided a less-energy-consumption path for H_2_S oxidation on the electrode surface. The sensor alleviated electrode poisoning owing to its antifouling properties. The modified sensor converted the fouling-subtle surface to an autocleaning surface, an ideal platform for constant examination (see Figure 2B). The *o*-phenylenediamine-RGO-MoS_2_ electrode joined with AM presented a DL of 10 nM. The unification of the above-mentioned properties resulted in decent selectivity. The designed method was practically applied to monitor H_2_S release in *E. coli* for up to 5 h. The technique could be extended to detect H_2_S in supplementary living systems and be used as an analytical device in biomedical applications. Nanocasting makes it simple to build ordered porous materials by using solid templates like tightly packed colloidal crystals. Using in situ created NiO nanoarrays as a template, it is currently possible to fabricate SnO_2_ nanoarrays with porous netlike structures on a chip. As-prepared SnO_2_ nanoarrays exhibit exceptional selectivity, stability, and the capacity to respond linearly to H_2_S in a wide concentration range (0.4–80 ppm), in contrast to the poor stability of NiO nanoarrays’ response to H_2_S. Due to the unrestricted passage of gas molecules to the porous netlike architecture, SnO_2_ nanoarrays exhibit a significantly higher and faster H_2_S response than their dense film counterparts [77].

#### 3.1.2. H_2_O_2_ Detection

**Figure 3:**
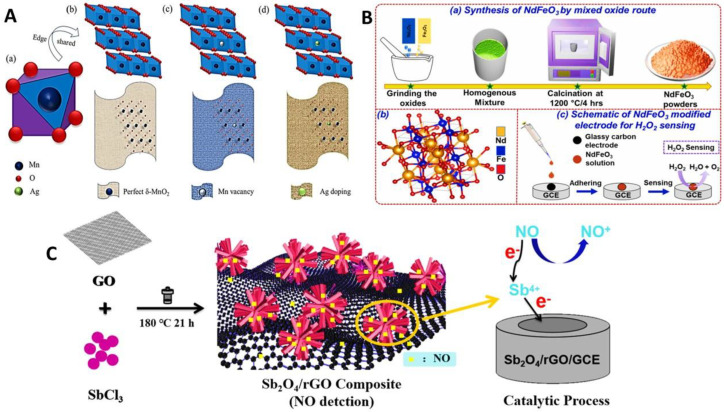
(**A**) Schematic representation of the [MnO_6_] octahedral unit’s fundamental structure (**a**), perfectly aligned [MnO_2_] nanorods (**b**), Mn vacancies in [MnO_2_] nanorods (**c**), and Ag doping in [MnO_2_] nanorods (**d**). Reprinted with permission from [79]. (**B**) NdFeO_3_ fabrication using a mixed oxide method in (**a**), and its molecular structure, and (**b**) modified NdFeO_3_ electrodes can be used to detect H_2_O_2_, (c) GCE modification for the detection of H_2_O_2_, Reprinted with permission from [87]. (**C**) Schematic of Sb_2_O_4_ nanoflowers grown on RGO and EC detection of NO. Reprinted with permission from [91].

H_2_O_2_ is a partially reduced metabolite of O_2_ with a varied range of biological and pathological effects inside active cells liable on the concentration, timing, and location of its production. Classification of H_2_O_2_ cellular functions needs the extent of its concentration, specifically in other O_2_ metabolites with three-dimensional and sequential reliability in living cells. H_2_O_2_ is involved in various therapeutic courses, i.e., antibacterial resistance, wound healing, stem cell production, and neuronal protection. An abundance of H_2_O_2_ release employs lethal effects on cellular sections that lead to diabetes, aging, cancer, and brain diseases [78]. Cytotoxic H_2_O_2_ is a constant in our life, even in the middle of the current pandemic COVID-19. Frontline coronavirus fighters may swiftly be cleaned up and used using H_2_O_2_. Recently, a nonenzymatic Ag@δ-MnO_2_/G graphene nanocomposite supported by Ag doped δ-MnO_2_ nanorods was reported for the subtle detection of H_2_O_2_. The δ-MnO_2_’s low electrical conductivity and Ag NP aggregation were improved by the ternary nanocomposite (as shown in Figure 3A) [79]. Additionally, δ-MnO_2_/G demonstrated an asymmetrical surface as well as several functional moieties for doping more Ag atoms, hence modifying the NCs electrical properties. Ag@δ-MnO_2_/electroactive surface area greatly improved as a result, and it was discovered that it had outstanding catalytic activity for the reduction of H_2_O_2_.

A simplistic technique developed an EC sensor based on 3D GR frameworks from Glu sugar with the assistance of melted salt for subtle nonenzymatic detection of H_2_O_2_. The electrode materials were characterized by scanning and transmission electron microscope (SEM, TEM) that showed thin layered GR formation. Atomic force microscope (AFM) analysis showed a 1.5 nm size of GR frameworks that further confirmed the GR structure. The EC investigation revealed that GR-modified electrodes possess a 0.143 cm^2^ EC active surface area (ASA) higher than the unmodified glass calomel electrode (GCE). The EC displayed amazing EC activity toward H_2_O_2_ reduction with a low (0.032 µM) detection limit (DL) via AM technique. Furthermore, the EC sensor detected trace amounts of H_2_O_2_ released from turmeric cells [80]. Singh and coworkers [81] reported a low-cost catalase mimicking aptitude of brominated GR via EC detection of H_2_O_2_. The modified GCE exhibited superior response toward H_2_O_2_ reduction. The fabricated sensor exhibited the double mimicking aptitude as catalase and peroxidase under different parameters by employing cyclic voltammetry (CV). The mechanism for H_2_O_2_ sensing by the formation of (HO_2_˙) is enabled by brominated GR/GCE. The developed EC sensor showed 100 µM–10 mM linear range (LR) and 0.063 mM DL via differential pulse voltammetry (DPV). Their sensor showed highly reproducible and selective results against other bioanalytes. The room temperature storage of the fabricated sensor abated the specificity and problem linked with normal enzymes. Similarly, an indium tin oxide (ITO) electrode based on Nafion (NF)/GO@Au NPs nanozyme was reported for EC sensing of H_2_O_2_ where tetramethylbenzidine (TMB) was used as an oxidoreduction mediator. Throughout the catalytic reaction, the TMB was oxidized to form its oxidation product, which produced a blue color (for spectrophotometric analysis) and generated an EC signal. In the DPV results, the TMB peak current showed an unbending connection with increasing H_2_O_2_ concentration in the LR of (10 nM–10 mM). The recorded DL of 1.9 nM was much lower than obtained with the spectrophotometric method [82]. Similarly, a non-enzymatic EC sensor was reported by combining metal/polymer and GR NC for H_2_O_2_ sensing. The fabricated material (AgNPs@RGO@PANI NC) was characterized by SEM, TEM, X-ray diffraction (XRD), and X-ray Photoelectron Spectroscopy (XPS) that confirmed the successful deposition of Ag NPs on RGO and the presence of other elements in the material. The EC properties of AgNPs@RGO@PANI/GCE exhibited 0.2 cm^2^ (ASA). The EC peak current showed linear relation with increasing H_2_O_2_ concentration that indicated that PANI incorporation on GR preferred the electron transfer to H_2_O_2_ during EC reduction. The main part of PANI was to enable increased surface area specific for interaction of target analyte consequently electron transfer kinetics enhanced during its EC reduction as follows [83]:

H_2_O_2_ + AgNPs → OH_ads_ + OH^−^ (3)

OH_ads_ + AgNPs + H^+^ → H_2_O + ½ O_2_ (4)

The modified GCE presented outstanding EC performance for H_2_O_2_ reduction. The (AM) response displayed an LR of (0.01–1 mM) and 50 nM DL due to the straight electronic contact of Ag NPs with the nitrogen atom of PANI support on the RGO sheet. High sensitivity and selectivity in interfering bioanalytes (Glu, DA, AA) were achieved for H_2_O_2_ sensing. Artificial enzyme mimics have grown significant attention for sensing platforms due to their better catalytic activity and high stability. 3D GR-based materials were created in order to maintain the inherent attributes of 2D GR while improving additional characteristics, such as specific surface area, porosity, tensile strength, and so forth. The one-step, efficient method of completely adorning crumpled GR with copper chloride hydroxide hydrate nanoparticles was demonstrated. The copper-based NPs were immediately associated with the crumpled GR because the carbon NSs served as a platform for the nucleation and development of the nanoparticles. With only a little modification in the quantity of copper salt precursor, the efficiency of the H_2_O_2_ sensor depends on the accurate distribution of NPs on the GR. Crumpled graphene and copper-based nanoparticles collaborate to achieve high sensitivity, linear H_2_O_2_ response curves, selectivity, and repeatability. The revised electrodes were easily maintained in dry and ambient settings. The composites of crumpled graphene and copper chloride hydroxide hydrate nanoparticles for H_2_O_2_ detection exhibited a longer endurance than enzymatic sensors [84]. Alizadeh and coworkers [85] developed a microfluidic device-based EC sensor by taking advantage of the intrinsic enzymelike properties of cerium oxide NCs. The cerium oxide NCs unveiled oxidase, catalase, and peroxidase triple enzyme simulated activities. These properties were controlled by regulating the pH. The NC’s working mechanisms were examined by electron paramagnetic resonance spectroscopy, which showed that peroxidaselike property originated from the production of (^•^OH) radicals. The catalase activity caused the breakdown of H_2_O_2_ to O_2_. The modified electrode exhibited a commendable catalytic response toward reducing LR of (100 nM–20 mM) with 20 nM DL by using the chronoamperometric (CAM) technique. The developed device was employed for real-time detection of H_2_O_2_ release from PC12 cells. A flexible EC sensor based on laser-scribed GR and Ag NPs was developed to enhance catalytic response towards H_2_O_2_. The fabricated sensor was verified under constant bending and various interfering molecules and H_2_O_2_ were detected in milk samples [86]. NdFeO_3_ has good thermal stability and is nontoxic, making it an appropriate candidate for EC sensing systems. In the current study, solid-state reactions carried out at a higher temperature are used to create multiferroic NdFeO_3_ particles. The orthorhombic symmetry of NdFeO_3_ crystallization is confirmed by structural research (see Figure 3B), [87]. The GCE was covered with single-phase room temperature NdFeO_3_ multiferroics to use a three-electrode EC setup to detect H_2_O_2_. When the concentration of H_2_O_2_ increased from 1–10 mM with a DL of 0.87 µM, the CV responses revealed an upsurge in the peak values. The sensors showed excellent selectivity towards H_2_O_2_ in the presence of HCl, H_2_SO_4_, KCl, and NaOH in milk samples and good sensitivity for measuring H_2_O_2_ in those samples. Zhou and colleagues [88] reported an EC sensor by incorporating NiMn-layered double hydroxide (LDH) on GO. The sensor was fabricated via a hydrothermal approach where first, metal cations were adsorbed on a GO sheet that later undergoes further nucleation growth to form a homogenous structure. The dangling O_2_ moieties GO surface served as nucleation cores for LDH deposition, which prevented the aggregation of NiMn-LDH/GO NSs. The as-prepared sensor exhibited notable EC performance by CAM for H_2_O_2_ reduction with high sensitivity and 4.4 μM, DL. One more sandwich-type EC sensor was constructed on a screen-printed C electrode (SPCE) via EC deposition and coreduction scheme. The AM responses of PtNP/RGO–CNT/PtNP/SPCE were evaluated that showed good EC performance compared to other control materials and a LR of (25 μM–1 mM) was achieved. Last, the immediate detection of H_2_O_2_ release from LNCaP cells was recorded proving its applicability for in vitro analysis in pathological specimens [89]. Furthermore, bimetallic Pt/Pb NCs integrated with GR-based EC sensors were reported for sensitive detection of H_2_O_2_ in live cells and a neutral solution [90]. The prepared PtPb/GR NCs displayed superior EC activity toward H_2_O_2_ reduction in a half-cell test with (2 nM–2.5 mM) LR and a low DL of 2 nM. The sensitivity of this modified sensor was ≥12.7 times as compared to the commercial Pt/C sensor for H_2_O_2_ detection. The densely packed EC active sites of Pt/Pb nanoplates and the synergetic effect among Pt/Pb and GR appeared to be the key features in contributing to the higher EC performance. The sensor was used for instantaneous detection of H_2_O_2_ secreted from Raw 264.7 cells. Their study highlighted the role of structural composition and fine-tuning of metallic NPs in developing highly sensitive nonenzymatic EC sensors.

#### 3.1.3. NO Detection

Since the finding that (NO) was the endothelial derivative easing factor, information on the accurate range of NO action in living functions has swiftly expanded. NO plays a significant role in immune system resistance, vasodilation, wound healing, neurotransmission, and cancer biology [92]. Owed to its understanding of biological systems, it becomes clearer that it functions in a concentration-dependent way. Low levels of NO cause anti-inflammatories by overwhelming the production of T helper cells, whereas high concentrations provoke a strong proinflammatory effect in the existence of pathogens. Therefore, the particular quantification of NO concentration in living cells is important for the timely diagnosis and treatment of various diseases. In this regard, a sensitive EC sensor based on Sb_2_O_4_/RGO nanoflowers was developed by a simple hydrothermal approach for NO detection released from living cells as shown in Figure 3C, [91]. The fabricated sensor solved potential problems, fast decay, slow electrode kinetics, and instability caused by electrode poisoning for NO detection. The Sb_2_O_4_/RGO/GCE showed greater EC performances owing to fast electron transfer enabled by Sb^+4^, greater charge transportation offered by extremely conductive RGO, and unique nanoflower morphology provided 3D more exposed active sites for NO to easily reach the electrode interface with high mass transport. The EC sensor exhibited high sensitivity, wide LR, and low DL of 3.9 nM. The real-time tracking of NO released from normal and cancerous cell lines by ACh stimulation was analyzed, and results revealed that cancer cells released NO five times than that of normal cells. Since the ubiquitous NO signal molecule is unstable both in vivo and in vitro, it needs to be found immediately and directly in order to provide information. In view of this, a recent method using a CPE modified by drop casting for rapid EC detection of NO at 0.72 V (vs. Ag/AgCl) was given. A NC made of Nafion, GR-NR, and Au NPs was fabricated as a modifying solution. At room temperature, voltammetric tests were run. The acquired results for the analytical signal showed reliable linear ranges of 0.39–105 µM based on DPV measurements. Research investigating the impacts of physiological interferences also yielded negligible results. Last, the sensor worked well for monitoring NO release. According to the results, the novel sensor provided a quick and simple method to identify low NO concentrations in liquids using direct voltammetry [93].

Similarly, an EC sensing platform based on ITO electrode-modified iron phthalocyanine (FePC) grafted on nitrogen-doped GR with NF and poly-L-lysine (PLL) layers was designed for real-time detection of NO. The FePC was deposited on N-GR by a noncovalent association that induced additional active spots for the catalytic redox reaction. The modified electrode showed distinguished EC performance toward NO oxidation with LR of (0.18–400 μM) and 180 nM DL. The layered assembly of NF and PLL further enhanced the capacity of resistive interference besides the electrode interface’s physiological compatibility. The ITO-based electrode’s flexible strategy offered an additional precise cellular EC sensing system that instantly captures current signals after NO is released from normal skin cells [94]. A dual EC sensor for the determination of nitrite NO_2_^−^ and NO based on the simple strategy of the mono layer of Au NPs self-assembly on sulfur-doped GR was presented [95]. The structural properties were evaluated by SEM, TEM, XRD, and XPS analysis. The EC responses showed that nano Au is well exposed for faradic progressions due to the dominated Au (111) crystal planes as well as stable, reusable for selective detection of biologically and ecologically essential nitrogen oxides. The AuNP/SGR/GCE demonstrated the sensitive EC quantification of NO and NO_2_^−^ with low DL (9 nM and 3 nM) individually. The fabricated sensor detected NO in natural water samples with high selectivity and reproducibility. Yoon and colleagues [96] developed a novel EC sensor based on NH_2_-MoS_2_/GO/myoglobin (Mb) for NO detection. The NH_2_-MoS_2_ hybridized with GO by electrostatic interaction and the Mb was immobilized on this hybrid. TEM, UV-Vis, and AFM evaluated the structural and morphological evaluation of the prepared sensor. The modified GCE exhibited enhanced EC performance recorded by AM *i-t* curves for NO detection as compared to simple MoS_2_/GO/Mb/GCE. The sensor detected NO with high specificity in presence of other interfering molecules with low 3.6 nM DL. The strategy of metal oxide NSs coupled with GR as the backbone can increase the electron passage from the inside. Simultaneously, the external layer can smooth the ion transfer, bringing assistance to applications in plentiful EC technologies. Due to the close relationship between charged elements of semiconducting metal hybrids and electromagnetic waves, which progressively move the interface charges, the formation of noble metals on MOs can effectively influence their characteristics. Polarization is produced by this restructuring of the connecting surface charges and could improve the electrocatalytic abilities. In this regard, an EC sensor based on bimetallic Co_3_O_4_@Pt NCs merged on RGO NSs for NO oxidation was developed. The structural properties were evaluated by SEM, Raman spectra, and EDS mapping that confirmed all these elements in the material. The RGO/Co_3_O_4_@Pt/GCE shifted the oxidation potential towards negative with improved peak current for in situ generated NO. The synergy of two metals and GR displayed good EC performance with 1.7 μM DL and high selectivity in the existence of a 100-fold concentration of other bioanalytes [97]. A high-performance NO sensor was fabricated by Zn- dithiooxamide framework derived from ZnO NPs. The ZnO NPs were immobilized on PTTA-RGO NCs to enhance the stability of porous ZnO. The structure of prepared two different-sized NPs was evaluated by TEM and FESEM. The EC studies of modified electrodes revealed that small-sized ZnO NPs presented enhanced electrocatalytic response toward NO sensing with a low DL of 7 nM. The extremely spongy surface of ZnO offered a high surface area for electrolyte and enhanced ion-charge transfer that proficiently catalyzed the NO reduction. The developed sensor was applied to detect NO released from normal (HEK-293) and cancer (PC 12) cells. The EC performances showed a higher AM current response was recorded with cancer cells than normal cells following previous studies [98]. Developing EC sensors with higher sensitivity that synchronously hold good biocompatibility for NO tracking at the cell level is a great challenge. To solve this problem, Liu and colleagues [99] developed a biomimetic and reusable micro EC sensor based on 3-aminophenylboronic acid and metalloporphyrin functionalized RGO for NO detection. The hybrid NSs unveiled improved exposed active sites that enhanced the sensitivity of the sensor to the nM range. The coupling of 3-aminophenylboronic acid offered good cytocompatibility and reversible sensitivity that allowed hands-on reusability of the sensor. The modified ITO electrode exhibited notable AM response toward NO oxidation with 5 nM DL and real-time tracking of NO released from endothelial cells cultured on the surface of the sensor. In addition, owing to the transparency of the sensors it was coupled fluorescent imaging systems. The developed sensors could be integrated into microfluidic or implantable devices for NO sensing at trace levels.

### 3.2. Neurotransmitter Detection

#### 3.2.1. Detecting Dopamine (DA)

**Figure 4:**
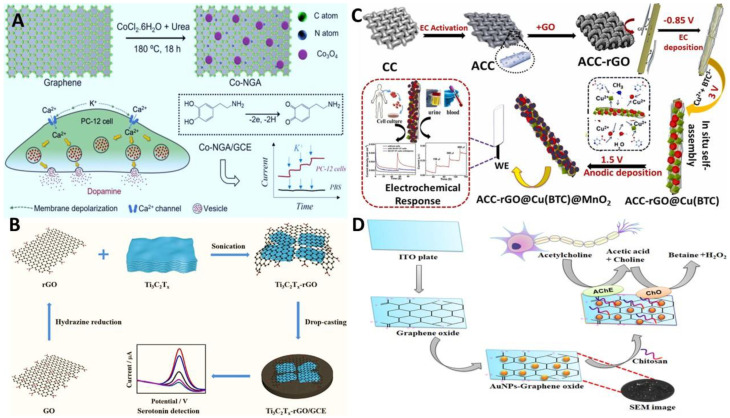
(**A**) Drawing of Co-NGA and Co-NGA/GCE used to detect DA secreted by live cells. Reprinted with permission from [108]. (**B**) Synthesis of Ti_3_C_2_T_x_-RGO NC and its EC response for ST detection using GCE. Reprinted with permission from [125]. (**C**) Schematic of ACC-RGO@Cu(BTC)@α-MnO_2_ based flexible electrode prepared by anodic-induced electrodeposition and EC detection of Ach from biological matrices. Reprinted with permission from [127]. (**D**) Depiction of Ach detection mechanism on the Surface of ITO-Au NPs-GO modified electrode. Reprinted with permission from [129].

DA is an extensively studied monoamine neurotransmitter that plays a vital role in various biological courses. It is primarily involved in hormone release and communication responses. Low levels of DA in human serum lead to Parkinson’s disease a neurodegenerative disorder [100], while increased DA levels may cause schizophrenia, euphoria, and failure in energy absorption. Immediate and precise detection of DA is required to predict, diagnose, and treat common brain diseases. Among the several diagnostic methods, EC transducer-based approaches, being affordable, sensitive, and transportable, have been used widely for DA detection [101]. DA structure contains one benzene ring, OH and NH_2_ functional groups that can form a π bond with GR. This π stacking mostly occurs due to π–N^+^H_3_, π–π, and π–CH interactions. When GR-modified electrodes are used, the NH_2_ group on DA becomes positively charged at pH ≥ 5. So, positively charged DA molecules attract negatively charged groups on GR via strong electrostatic forces. DA generally undergoes double EC transfer with a free radical polymerization reaction and converts to melanin during an EC reaction [102]. Li et al. [103] reported a Cu(II) polymerized 2-amino-5-mercapto-1,3,4-thiadiazole (AMT)-based novel enzyme mimetic EC sensor. Afterward, NH integrated with RGO by π–π stacking interface as an effective mimetic enzyme for DA in actual samples. The RGO-(Cu-AMT) NCs presented substantial enzyme mimetic catalytic response due to fast electron transfer between the electrode and analyte surface. The EC performance of the biomimetic electrode showed high sensitivity and broad LR when applied for DA detection in human urine samples. However, frequently used sensors suffer from interference of AA, low sensitivity, and meager flexibility. To circumvent this issue, a flexible sensor was developed using polyolefin substrate and self-assembled GR [104]. The AA interference was cut down to the lowest by reducing the EC resistance and diffusion current. The EC performance revealed that Au electrodes synthesized on polyolefin films display a small charge transference resistance of about 20 Ω with high sensitivity of (7.8 μA μM^−1^) for DA oxidation 300 times higher than that of silicon electrodes. Similarly, a simple and low-cost EC was designed using xylan as a carbon source. Carbon quantum dots prepared by xylan was used as a reducing agent in the fabrication of Ag@CQD/RGO. The modified GCE showed notable EC performance in terms of high selectivity as the conductivity of the electrode was boosted by electrostatic interaction flanked by carboxyl and amine functional groups of GR and DA, respectively. This sensor-based extended the application of polysaccharide-based EC sensors for neurochemicals detection [105]. Our group reported an EC sensor for DA detection as an early Parkinson’s disease diagnosis tool. The sensor was fabricated by the periodic assembling of positively charged layered double hydroxides (LDHs) NSs with negatively charged single layers GR by thoroughly optimizing numerous parameters in a particular cofeeding mode. Due to improved intercalation ability of LDHs and high surface area with plentiful active sites being harvested as a result of the direct adjoining of extremely conductive GR to semiconductive LDHs layers, decent electrocatalytic performance in DA detection was demonstrated. The UA, and AA interference was effectively eradicated by modifying the electrode with NF. Moreover, the sensor was practically employed for DA released from a live human nerve cell line after stimulation. The sensor based on periodic stacking of 2D charged NSs opened a new prospect in exploring vertically influenced artificial 2D NMs and presented an exciting application potential in biosensing platforms and pathological diagnostics. In a recent study, a mixture of two different layered materials RGO NSs and WS_2_ separated by Fe_3_O_4_ NPs was fabricated for the detection of DA using microwave irradiation. A simple, affordable, high-yielding, and quick reaction time approach was used to create the heterostructure RGO-WS_2_@Fe_3_O_4_ nanocomposite. The phase development, structural properties, and surface morphology of the NC showed that the Fe_3_O_4_ NPs positioned between the separated layers of RGO and WS_2_ NSs inhibited NP agglomeration and prevented the 2D layered materials from becoming stacked once more. The DL of the RGO-WS_2_@Fe_3_O_4_ sensor was 2.74 µM [106]. As a result, it was envisaged that a cost-effective microwave-assisted method for RGO corresponding with other inorganic coated materials to develop EC devices for the determination of biotic and pharmacological samples can be established. By electroplating copper nanocubes on top of single-layer and multiple-layer GR, a comparable copper nanocube-based EC sensor for the detection of dopamine from plasma samples was developed. In order to produce cubic-shaped Cu NPs on top of GR, the copper electrodeposition technique was carefully reviewed and optimized, increasing the sensor’s active area. The CuNCs-Gr/SPCE nanozyme sensor was EC assessed by EIS and the reaction to DA oxidation was investigated using the DPV method. In the existence of other neurotransmitters, the developed sensor showed remarkable selectivity for DA. The reliability of the approach was demonstrated by the 95–100% recovery for the exposure of DA in human blood plasma samples [107]. Real-time monitoring of DA in living cells is essential for research and biological diagnosis of numerous central nervous system illnesses. The fast and sparse nature of DA release from cells necessitates the use of an analytical strategy that is highly responsive to DA. This paper presents a unique nitrogen-doped graphene aerogel/Co_3_O_4_ nanoparticle modified electrode (Co-NGA/GCE)-based EC sensing device. With 12 nM of DL, which was less than most of the described approaches, the Co-NGA/GCE conducted higher DA detection. This was made possible by its effective conductivity, high surface area, and catalytic activity. It was demonstrated that the Co-NGA/GCE was capable of realizing the monitoring of DA released by living cells in response to K^+^ stimulation see Figure 4A [108]. The Co-NGA/GCE is expected to not only be a perfect tool for DA assay but also to provide a strong alternative to pathogenesis research and medication screening for disorders of the nervous system. In order to track the dynamics of chemical neurotransmission, in vivo EC measurements need to have a quick temporal resolution. The most widely used EC technique for detecting swift changes in neurotransmitter levels in the brain is fast-scan CV [109,110]. Carbon nanospikes (CNSs) and CNTs on wires and CFs have been reported recently. These CNSs showed a rough surface shape with increased surface area, as well as a high number of graphitic flaws, which boosted EC performance. CNS electrodes provided a higher electron transmission rate and increased surface adsorption due to the presence of more electroactive edge-plane positions. Because of higher adsorption at CNS electrodes, cation neurochemicals performed better in fast scan CV than anions. CNSs provided a low-cost and upfront approach for fabricating homogenous coatings because they were created without a catalyst layer [111]. Chang and Venton [112] recently examined dip coating, drop casting, and electrodeposition procedures for coating salable GO right on carbon fiber microelectrode. Drop casting produced huge clusters that provided noisy signals and delayed rise times, but dip coating fabricated minute GO coating. Electrodeposition with CV boosted the current for DA, was the most reproducible, and had the least noise when compared to the other two coating techniques. The modified electrode boosted the DA oxidation peak with fast-scan CV with 11 nM detection limit. To show their effectiveness in tissue, the optimized electrodes were utilized to detect electrically activated DA in brain slices. Similarly, GO/PEDOT modified CFE was employed for in vivo DA detection. By carefully examining DA transient function in real time, it may be possible to gain a better understanding of DA functionality without sacrificing electrode kinetics. Electrodeposited PEDOT/GO layers offered excessive tunability. By adjusting the deposition size, current, and oxidation state of GO, coatings could be customized to any particular electroactive analyte [113]. Although GR fibers have been employed in the literature for a variety of purposes, there is a dearth of research on using them as microelectrode-sized neurochemical microsensors. The GR fibers fabricated recently with a typical diameter of 20 µm to 30 µm, and fast scan CV was used to characterize both GO and RGO fibers. An efficient, one-step, dimension-restricted hydrothermal method was used to synthesize neat GR fibers. Carbon-fiber microelectrodes, and more recently, CNT-fiber electrode, have been employed for fast scan CV detection; nevertheless, the homogeneous functionalization and direct control of the 3D surface structure of these materials are still limited [114]. The reported sensor demonstrated notable improvements in electron transport, fouling resistance, redox cycling, and frequency independent behavior when compared to standard carbon fiber microelectrodes, highlighting their tremendous value as biosensors.

#### 3.2.2. Detecting Serotonin (ST)

5-hydroxytryptamine commonly known as ST is a vital monoamine neurochemical present in brain for regulating temper, sleep, disgorgement, sexuality, craving, and pain [115,116]. The unbalanced ST concentration is associated with numerous conditions, including sadness, anxiety, hemicrania, and natural aging. This suggests that it is crucial to detect ST for wellbeing and medical treatment [117]. Given outstanding catalytic properties, GR’s hybridization with metal alloys has yielded exciting and extensive applications. Thanh and coworkers reported a high-quality nanosensor fabricated by Au-Ag alloys encapsulation on GR NSs using an electroless deposition method for ultrasensitive detection of ST. The NHs were obtained in the desired range with good reproducibility and free from external stabilizers, reducing agents, and binders. The modified electrodes exhibited first-rate EC performance with high sensitivity, wide LR, and lowest DL toward ST oxidation [118]. Likewise, two layers of the sensing interface was developed for serotonin recognition based on RGO/polyaniline (PANI) NCs and molecularly imprinted polymers (MIPs) engrained AuNPs. The RGO/PANI NCs were prepared via the electrodeposition method. Where protonated anilines were originally attached to the RGO sheets by electrostatic adsorption and then the RGO/PANI film was designed on the bare electrode via CV technique. The AuNPs@MIPs membrane was applied on the modified electrode by continuous potential technique in the presence of ST. The obtained sensor displayed notable selectivity toward ST against the interferences instigated by AA and UA. Moreover, the EC biomimetic sensor was practically employed for ST detection in serum samples [119]. Mahato and colleagues [120] have developed a label-free sensor based on Au NPs decorated with GR for ST oxidation. The sensor was optimized for EC performance evaluations that showed admirable diagnostic performance with the wide LR from (3 µM–1 mM) with 3.87 nM DL. The proposed EC sensor was then employed for ST detection in human serum, blood, and live cells that showed superb recovery rate of up to 98%, which is suitable for several medical applications. Additionally, a GO/chitosan-based immunosensor was designed for highly selective EC oxidation of ST. The carboxylic groups of GO were used for covalent bonding with amine groups of antibodies to ensure the biocomponent immobilization on the electrode surface. The developed sensor was optimized using various EC techniques and the modified sensor displayed 10 nM lowest DL for ST detection. Furthermore, the practical application of the developed sensor was evaluated by SR detection in various biotic matrices (serum, saliva, urine, and tears) in presence of biological interferences [121]. An EC sensor constructed on the self-assembly of Ag_2_Se NPs on the RGO surface via the probe sonication method was reported for ST determination. The EC ST oxidation mechanism on RGO-Ag_2_Se/GCE surface involved two-electron and proton transfer that converted to ST quinone imine. The EIS plot evaluation confirmed the high and significantly low charge transfer resistance (R_ct_) owed to the synergistic effect among RGO and Ag_2_Se. Therefore, the modified GCE permitted fast electron dispersion ST detection [122]. Cobalt oxide (Co_3_O_4_) cubes integrated with RGO were fabricated by a simple hydrothermal approach. The crystal assembly and morphological evaluations were conducted via FESEM, Raman, and XRD analysis. The EC performance evaluation of the fabricated RGO/Co_3_O_4_ sensor revealed good electrocatalytic aptitude towards ST as a depression biomarker. The AM investigation of the modified sensor recorded the DL of 100 nM with a wide dynamic LR of (1–10 μM). Similarly, the sensor presented good anti-interference aptitude in presence of various interfering molecules (UA, AA, DA) [123]. t is predicted that the above-mentioned reports will hold a diverse display of applications in bioelectronics, biosensors, primary disease diagnostics and medicinal chemistry. One technology that shows promise for creating NMs is atomic layer deposition. NMs can have their thickness and homogeneity accurately regulated. In order to achieve the best combination interface, homogenous Ni NPs were coated on RGO via atomic layer deposition. The results showed that the Ni NPs may occupy some of the RGO’s active surface, producing an NC of Ni-RGO with a high specific surface area and high conductivity. The electron transport flanked by the electrode surface and the solution was accelerated by the Ni NPs-RGO improved electrocatalytic activity for ST. As a result, the modified Ni NPs-RGO/GCE sensor was to detect ST by DPV. By showing an inclusive linear range of 0.02–2 µM with detection of 0.01 µM for ST, the Ni-RGO/GCE improved the current responses. For real sample detection, the Ni-RGO/GCE showed good stability, selectivity, and anti-interference characteristics. These results showed that the Ni-RGO NC may be produced successfully via atomic layer deposition. Ni NPs-RGO NC has the potential to be used in sensors and catalysis because of its distinctive features [124]. ST in human biofluids can currently be detected using a new EC sensor based on Ti_3_C_2_T_x_-RGO/GCE see Figure 4B [125]. Hydrazine reduction and self-assembly were used to create the Ti_3_C_2_T_x_-RGO nanocomposite, which was then studied using SEM, TEM, XRD, and Raman spectroscopy. In addition to reducing the restacking issue with Ti_3_C_2_T_x_ NSs, the produced Ti_3_C_2_T_x_-RGO NC offered a significant surface area and efficient active sites that enhanced the EC performance of the NC. For the detection of ST, the sensor based on the Ti_3_C_2_T_x_-RGO NC demonstrated great sensitivity, high specificity, and outstanding stability. Additionally, ST in human blood plasma was effectively measured using it, and the outcomes fell within the expected range. The ST detection technique presented in this study is anticipated to be used for the early detection and treatment of ST-related disorders.

#### 3.2.3. Detecting Acetylcholine (Ach)

The neurotransmitter acetylcholine (Ach), which was initially identified in the central nervous system, transmits impulses to various cell types. Memory, attention, and learning are just a few of the dynamic roles Ach and its metabolite choline (Ch) play in the brain. Meanwhile, loss of neural communication and inflection are associated with Ach, which causes various nervous ailments including Alzheimer’s, Parkinson’s disease, progressive dementia, and schizophrenia. A quick, sensitive, and accurate detection method is crucial in clinical applications to comprehend the physiological elements of nerve illnesses brought on by a drop in Ach concentration and their therapy. A sensitive EC sensor based on Fe_2_O_3_/RGO/PEDOT/FTO electrode was reported in this regard. Through quantitative and qualitative evaluations, the physical characteristics of the created NSs were further examined. The EC performance of the modified electrode displayed outstanding analytical features including an inclusive LR, fair recovery, manifold reusability, substantial selectivity, and quick response with incredible stability up to 100 days [126]. Recently, our group reported a flexible WE by achieving EC control over the production of HKUST-1 crystals via an anodic-induced electrodeposition method. By using EC deposition of pyramidal-octagonal MOFs (Cu-BTC) on a highly conductive and flexible carbon substrate (activated carbon cloth, ACC) covered in RGO layers, the problem of regulated mass transport and progressive MOF dispersion was resolved. To increase the synergetic influence of ternary structure interfaces, α-MnO_2_ was added to ACC-RGO@Cu(BTC). The new ACC-RGO@Cu(BTC)@α-MnO_2_ constructed working electrode displays outstanding EC presentation for nonenzymatic detection of Ach with an eclectic linear range of 0.1 µM–3 mM, lowest DL of 5 nM, strong selectivity, and longstanding stability. Furthermore, the proposed sensing device was used to detect Ach efflux in real time from three different cell lines and biological matrices (see Figure 4C) [127]. Our research opens up the possibility of precisely organized MOFs with comprehensive capabilities and enlarged production techniques for developing flexible sensing devices by selecting nanoscale reaction centers.

Innovative methods based on cutting-edge nanotechnology are required to identify Ach in actual samples. A NC layer of MWCNT-MnO_2_ was used to cover an Au electrode to create the ACh biosensor. Ach esterase (AChE) and ChO were then coimmobilized over the electrode (RGO). The biosensor was evaluated and improved employing the cyclic voltammetry (CV) method. CV method was employed to analyze the EC aptitude of the modified electrode for Ach detection with the following reactions on the electrode surface.

(5) $$\mathrm{Ach}+H_{2}O\;\overset{\mathrm{Ach}\;\mathrm{esterase}}{\to }\;\mathrm{Ch}+\mathrm{Acetate}$$

(6) $$\mathrm{Ch}+H_{2}O+O_{2}\;\overset{\mathrm{Ch}\;\mathrm{oxidase}}{\to }\;\mathrm{Betaine}+2H_{2}O_{2}$$

(7) $$H_{2}O_{2}\;\overset{0.6\;V}{\to }\;O_{2}+2H^{+}+2e^{-}$$

The NCs deposition on the electrode surface showed an increased in current intensity owed to the improvement of EC active surface area compared to bare Au electrode. This phenomenon proved the enhancement of EC aptitude by improving sensor conductivity that exhibited a wide LR of (0.1–100 µM). The fabricated sensor also reported comparable sensitivity to high-performance liquid chromatography (HPLC) method toward Ach detection. Similarly, a bienzymatic sensor based on AchE/ChO for the immobilization on GR/Pt NPs/ITO electrode by electrodeposition method was designed to detect Ach in human serum samples. The EC presentation of the modified electrode was optimized at pH 7.0 by using EIS and CV techniques. The developed biosensor improved the analytical performance of the sensor in terms of incredible stability (4 months), fast response (4 s), and lowest (5 nM) DL, respectively [128]. Yet, as the presentation of the biosensor significantly depends on the interfacial architecture of NMs, the enzyme must be immobilized in a way that promises the conservation of its organic activity and the availability of all available active sites along with the working efficiency of the transducing element. A functioning electrode made of an enzyme-based AuNPs-GO NC was produced on an ITO@ glass plate as shown in Figure 4D [129]. The existence of AuNPs was confirmed by several characterization techniques, including UV-Visible spectroscopy, XRD, and SEM. Additionally, FTIR supported AchE-ChO enzymes’ attachment to GO functional groups. CV data demonstrated the working electrode’s ability to sense Ach concentrations between 100 pM–1 µM. It was discovered that 100 pM was the DL. The results are also supported by SWV plots, which displayed good anodic peak for different concentrations of Ach. Studies on interference revealed that the electrode had strong selectivity for Ach when compared to other frequent interfering agents, making it possible to use this electrode as a quick and accurate Ach biosensor. Additionally, the nanointerface detected Ach in biological fluids like serum. Previous reports presented the covalent bonding of biomolecules on the GR surface that possess some downsides, such as low reproducibility and the likelihood of disrupting the enzymes’ functionality, hence affecting the sensor performance. To eliminate this problem, Fenoy et al. [130] presented a GR-based field-effect transistor by electropolymerization approach. The voltammetric preparation of PABA (polymer linker) with amine functionalities allowed the simultaneous immobilization of AchE and enhanced the sensor’s pH response. The AchE-PABA-GR sensor displayed a swipe in the Dirac point toward negative in the existence of Ach because of enzyme-catalyzed hydrolysis, which provided real-time detection in the range of (5 µM–1 mM). Compared with other sensors, AchE-PABA-GR displayed ultrasensitive Ach detection urine samples and flow conditions. Their proposed approach presented a promising start for the development of sensors using voltammetric electrosynthesis, which is free from the need for chemical primers and provides high stability and particular control on film thickness. Therefore, the functionalization method could be expanded to the incorporation of other NMs or enzymes for hands-on sensing applications. Table 1 depicts recently reported EC sensors using GR for different bioanalytes.

### 3.3. DNA Sensors

**Figure 5:**
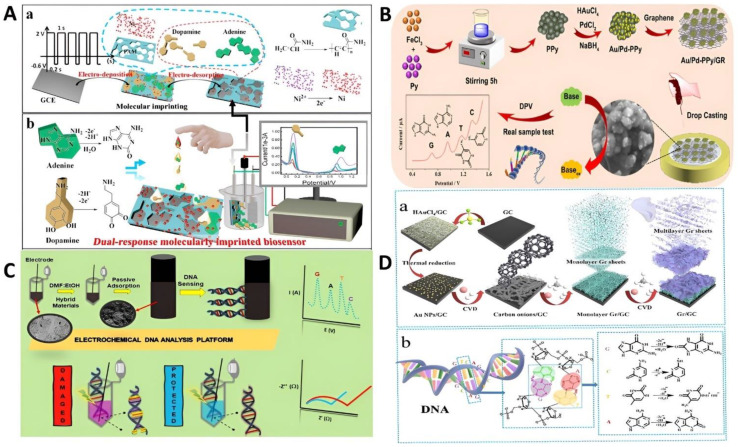
(**A**) Assembly technique of the dual-response MIP detection membrane for A and DA, (**a**,**b**) A and DA EC detection mechanisms. Reprinted with permission from [167]. (**B**) Synthesis of Au/Pd−PPy/GR/GCE and EC response for A, G, T, C detection. Reprinted with permission from [169]. (**C**) Fabrication of PtNPs/CB/RGO nanohybrid and EIS response for G sensing. Reprinted with permission from [171]. (**D**) Pictorial presentation of GR/GC fabrication process (**a**), interface between different DNA bases (A, G, T, and C) and their oxidation reaction (**b**). Reprinted with permission from [172].

Deoxyribonucleic acid (DNA) is a prominently active bioanalyte with a 3D double helix structure comprised of two chains consecutively in reverse directions. Owing to its major role in protein synthesis and genetic information DNA storage has attracted immense research interest in genome sequencing and other biology-related fields. Purines and pyrimidines are basic building blocks of DNA that play a fundamental role in neurotransmitter release, cell proliferation metabolic cofactors, and the cardiovascular system. DNA can be employed as a material to allow computation of diverse mechanisms on the probe surface, provide for self-assembly of elements, and enable them to attain the improved analytical response owing to the suitable longitudinal alignment of diverse biochemical species [159,160]. GR composites are desirable because, in addition to combining the advantageous optical, electronic, magnetic, and structural properties of NPs and GR that are not available in bulk materials, they also provide unique physicochemical properties and functions for use in bioapplications than any material alone [161]. Using EC detection techniques, the GR-based DNA biosensor makes it easier to identify different types of biomarkers. The EC method uses covalent bonds interactions, or the chemistry of N,N-(3-dimethylaminopropyl)-N′-ethyl-carbodiimide hydrochloride and N-hydroxysuccinimide chemistry to immobilize DNA on the GR surface. EC signals are generated when DNA is hybridized or oxidized. EIS, DPV, and CV, were used to quantify voltage and current shifts caused by a range of variables, such as changes in conductivity or electron depletion caused by oxidation or hybridization [162]. Circulating tumor DNA (CTDNA) is a vital biomarker for determining how well a patient will respond to treatment and is crucial for the early detection and prognosis of many types of cancer. It is impossible to quantify CTDNA precisely and specifically due to the low concentration of CTDNA in peripheral blood and the significant background of wild-type DNA. A novel 3D GR/AuPtPd nanoflower sensing platform was recently developed based on the efficient detection of CTDNA by the CRISPR/Cas9 cleavage-triggered entropy-driven strand displacement reaction (ESDR). This amplification necessitates sophisticated operating methods and strict reaction conditions and enabled the identification of small amounts of CTDNA. By combining the advantages of ESDR with those of site-specific cleavage by “gene magic scissors,” Cas9/sgRNA, they achieved high specificity for differentiating single-nucleotide mismatches as well as amplification efficiency [163]. In experiments with human serum, the proposed biosensor demonstrated good specificity and commendable performance. As a result, this pioneered technology established a new paradigm for effective CTDNA detection and has significant clinical and analytical potential. As compared to the above-mentioned techniques, EC sensors hold high sensitivity, rapid response, and ease of operation. Recently, multiple NC materials have been fabricated to detect DNA bases such as polyxanthurenic acid/RGO, iron hexacyanoferrate, and boron-doped CNWs [164,165]. Therefore, the advancement of the tractable way of sensing DNA bases is significant in life science and bioanalysis. Chem. adenine (A), guanine (G), cytosine (C), and thymine (T) are the four main bases of DNA. Various reports have focused particularly on genotoxicity and DNA impairment. Nevertheless, the development of a quick and multiplex system for the real-time detection of all DNA bases remains a challenge. Therefore, an Au-RGO/MWCNT/graphite NCs via a simple electrocodeposition method was fabricated for multiplex detection of G, A, T, and C, using SWV. The developed sensor was employed practically in plant and animal DNA samples in which C and T hydrolysis of 8 mol L^−1^ of acid was achieved. The EC performances of the modified electrode presented wide LR and good DL for the four bases separately with high selectivity in the presence of other bioanalytes. The reliability of the EC sensor was evaluated by comparison HPLC approach and a correlation of (103%) was recorded [166]. The preservation of the original morphology, robust adhesion to the collector, and a customizable structure are only a few benefits of in situ-prepared MIP membranes. Recently, a dual-response Ni-PAM-based MIP EC biosensor was developed to detect DA and AA simultaneously. First, an electropolymerized acrylamide (PAM) with Ni support was used to create a dual-MIP sensitive layer. This was the first description of a method for producing solid MIP films on the surfaces of various basic electrodes. Second, following thorough characterization, it was discovered that the dual-MIP sensor had significant electrocatalytic activity and a low charge transfer resistance of 8 Ω at the electrolyte/electrode interface. In addition, as seen in Figure 5A, the dual-MIP sensing electrode demonstrated considerably higher response sensitivity to A and DA as compared to the GCE [167]. The imprinted cavity is required to successfully prevent interference between the two analytes since the components of the imprinted membrane only exhibit intermittent electrocatalytic activity for DA and A. The quantitative detection model was discovered to be more reliable as a result of the sensor’s MIP detecting technique, which boosted the specificity for DA and A. The sensor’s suitability for the quantitative assessment of DA and A in different physiological fluids, including cells, blood, and urine was demonstrated by a number of quantitative detection models developed across a broad pH range. The developed method was anticipated to make it easier to build EC biosensors that respond to multiple targets and to reliably detect small bioanalytes with high selectivity. An EC sensor based on MnO_2_ and ionic liquid functionalized (IL-GR) bound to PDA membrane can detect G and A in blood samples. On modified electrode, G and A both go through a redox reaction. The EC performance of a GCE modified with PDA/MnO_2_/IL-GR was assessed using CV and DPV. The sensor-enabled the immediate and individual detection of G and A. The modified GCE was successfully used to analyze G and A in samples of spiked embryonic bovine fluid and mouse blood [168]. To store genetic information, the particular arrangement of DNA bases is necessary. To detect guanine G, A, T, and C separately and simultaneously, an Au/Pd-PPy/GR-based EC sensor was developed. The electroactive area of the bimetal nanomaterial (Au/Pd-PPy) was further increased, and the EC response was amplified by the GR incorporation. This improved the detection presentation of DNA bases. The EC sensor performance was decent, with well-distinguished current peaks at 0.976 V (A), 0.704 V (G), 1.348 V (C), and 1.164 V (T). The use of EC sensors in bioscience was demonstrated by the developed device, which was shown to be an incredibly sensitive and highly selective sensor for identifying the DNA bases in calf thymus DNA (Figure 5B) [169]. It was reported that a rapid and sensitive EC sensing platform based on bimetallic (Au-Pt NCs) distributed on RGO could detect G and A simultaneously using SWV. Au-Pt-RGO NC was synthesized by reducing GO and metal ions (Au^3+^ and Pt^4+^) in an aqueous solution at the same time. Under physiological pH, the designed Au-Pt NCs-RGO EC sensor with the optimized 50:50 bimetallic (Au:Pt) composition demonstrated exceptional electrocatalytic performance toward the simultaneous oxidation of G and A without the use of any enzymes or mediators. The sensing platform achieved DL of 60 nM for G and 100 nM for A, with good sensitivity and a broad linear range of 1 µM–0.2 mM. Some of the most frequent EC active interferents included AA, UA, and DA, but their effects were negligible [170]. The Au-Pt-RGO NC unveiled the potential for promising clinical diagnosis and biomedical applications, as confirmed by the proposed platform’s successful simultaneous detection of G and A in a denatured Salmon sperm DNA sample. Due to their reliable electroanalytical characteristics, hybrid materials based on carbon black have attained substantial attention in the expansion of EC sensors. In light of this, hybrid platforms based on PtNPs/CB (carbon black)/RGO were developed for label-free EC-DNA and DNA damage sensing. Utilizing the microwave-assisted reduction technique, various hybrids were effectively created by effectively impregnating Pt NPs. To advance the surface properties of pencil graphite electrodes, these materials were used to modify them. First, fish sperm DNA was examined using modified electrodes. Based on (G) oxidation by DPV, an investigation into the effects of various weight-to-weight percentage ratios of CB and RGO revealed that the hybrid material with a 50:50 ratio showed superior sensitivity and a low DL of 0.14 mg L^−1^. PtNPs/CB and PtNPs/RGO modified electrodes were also used in this comparison to make it more comprehensive. Additionally, the hybrid material modified electrode’s performance in electroanalytical tests based on (T) oxidation was examined. Later, AA was used in protection studies after EIS spectroscopy was used to successfully detect DNA impairment in the occurrence of Cu(II)/H_2_O_2_ reagents (see Figure 5C) [171]. The study emphasized the exceptional reproducibility and sensitivity of CB-RGO-based NP decoration.

Lei and colleagues [173] designed an eco-friendly EC sensor based on Cu-Ni@N, B-RGO using Glu as a reductant and stabilizer and linked it with an electrode for reliable and sensitive DNA purine bases (G, A) detection. The prepared NCs showed a significant synergy effect of high conductivity, specific surface area, and exceptional catalytic activity because of Cu, and Ni metals incorporation on N, B doped RGO surface. With the modified GCE, when employed to the investigation of DNA bases G, and A using DPV, the wide LR with DL of 0.11 μM and 0.13 μM were attained individually. With an A/G ratio of 0.80, the sensor was successfully used to detect A and G in calf thymus DNA. The innovative EC sensors are now possible thanks to the facetious synthesis technique and outstanding EC sensing capabilities of the NCs. The nanomechanical shift of practical 3D-DNA NS is vital for nanobiotechnological applications, for example, nanorobotics sensors and actuator devices. On this basis, a research group [174] modified the DNA tetrahedron strands with complementary sequences in a stem-loop structure, which responds to a peripheral stimulus due to attached ferrocene. The switchable device’s EC performance exhibited a decline in output signals under DNA hybridization because of the extended distance between the electrode and ferrocene interface. The EC performance of the nanodevice was also flexible under diverse ionic strength based on electrostatic exchanges amid the negatively charged DNA support and the species in solution, allowing modification and control of EC DNA sensors. Recently, GR sheets were successively grown on a GC substrate with the aid of Au NPs as catalysts to construct a novel carbon film. By examining variations in morphology, microstructure, and composition, it was possible to better understand how monolayer GR sheets form as well as the vertical development approach of multilayer GR sheets. For this function, which was based on the electrocatalytic activity of the carbon sheet itself, no additional delicate ingredients were required. The four DNA bases were used to evaluate the capability of the multilayer carbon film as an EC biosensor (Figure 5D) [172]. In a neutral electrolyte environment, the carbon film successfully identified G, A, T, and C with good DL limits. A-G, T-A, and C-T had peak potential differences of 164, 216, and 184 mV, respectively. This carbon sheet can be utilized as a basic electrode in the development of new EC sensors with high functionality and sensitivity, as well as to replace commercial GC, Au, and CPE sensors.

### 3.4. EC Immunosensors

**Figure 6:**
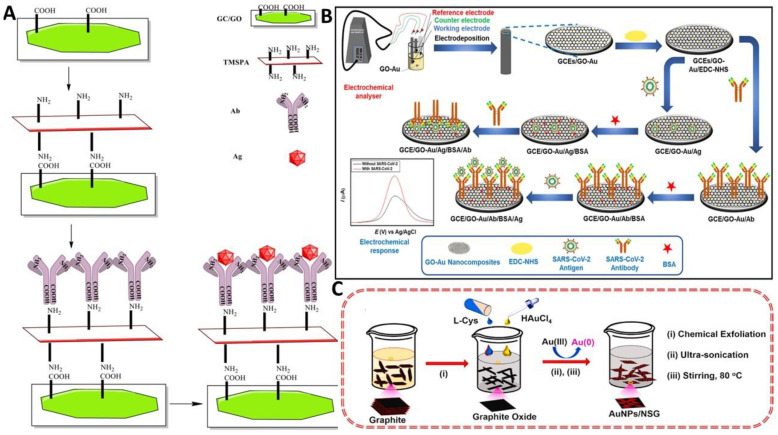
(**A**) The schematic of the Ag/Ab/TMSPA/GO/GCE fabrication process in steps. Reprinted with permission from [181]. (**B**) A flowchart showing the steps needed to make SARS-CoV-2 antigen and antibody immunosensors. Reprinted with permission from [185]. (**C**) Depiction of EC immunosensing platform made from in situ functionalizations of AuNPs/RSG. Reprinted with permission from [189].

Immunosensors are biosensing devices in which molecular recognition-based immunochemical reactions are coupled with transducers [175]. These particular interactions are employed to directly or indirectly detect immunochemical reactions. According to the immunoassay format (sandwich type, competition, or capture), the tagging of either the antibody or the antigen with signaling molecules enables the indirect technique to detect the immunological complex. Although electrochemical detection is an option, optical detection techniques (such as fluorescence chemiluminescence, electrochemiluminescence, and absorbance) are employed the majority of the time. In label-free direct detection technologies, the specific binding event between the antibody and the target analyte (the antigen) is monitored by a change or alteration in physicochemical properties. These direct detection techniques can also be used to obtain kinetic data on antigen–antibody responses [176]. Furthermore, immunosensors have been grouped according to their measuring principle, such as EC immunosensors (potentiometric, voltametric, capacitive, impedimetric, conductimetric and amperometric immunosensors), optical, piezoelectric acoustic, thermometric, etc. In an EC immunosensor, the target molecules are captured and detected using antibodies, which generate electrical charges for quantitative analysis [177]. Numerous studies have focused on the development and use of immunosensors linked to various types of detection, highlighting the key benefits of their construction processes [178,179]. Immunoreactions in EC transducers can change potential, ion concentration, capacitance, conductance, and impedance. Due to the fact that they are frequently compact, inexpensive, robust, have quick response times, and can be mass-produced, EC transducers are frequently used as immunosensors for a variety of applications. Due to their high molecular weight, immunoanalytical methods primarily have drawbacks (a number of analytes with interest in the environment, pharmaceuticals, and food have molecular weights below 1 kDa). It is important to choose the appropriate markers, which must have a strong affinity for binding antibodies, excellent sensitivity, constancy, and affordability. The Ab-Ag interactions are frequently not immediately reversible because of high affinity constant values. Immunosensors should ideally be able to continuously and selectively identify the target species [180]. Immunoglobulin G, or IgG, can be found in all bodily fluids. Immune response against bacterial and viral infections is triggered by a definite bond flanked by IgG and anti-IgG. For the detection and determination of antibody–antigen contacts, a new EC immunosensor constructed on (Ab/TMSPA (trimethoxysilyl)propylamine)/GO/GCE) was developed. This method makes use of (-COOH) on GO and 3TMSPA as shown in Figure 6A [181]. The monitoring of how an antibody interacted with various antigen concentrations used a change in impedance response. According to the findings, the electron transfer resistance increased as antigen concentrations rose. The EIS technique can efficiently imitate several biochemical and physical courses that occur on the electrode surface therefore it is frequently used for protein detection. The sandwich-type immunochemical assay similarly used the antibody (Ab2), which is frequently characterized by EC probes and NMs. The essential element of the EC sensor is the modified electrode surface, which recognizes the signal and transforms it into an electric signal like voltage, current, and resistance [182]. This output signal can be measured and evaluated to realize the quantitative or qualitative study of the target. An EC immunosensing platform for p53 protein was recently developed based on a triple signal extension approach using Au surface, which provided a high surface area for anti-p53 immobilization [183]. The Au NPs electrodeposited over P-Cys-GQDs improved the electron transference efficiency due to the synergy of P-Cys conductive medium and GQDs/GNPs as double magnification elements. The proposed immunosensor was applied for p53 detection in biological pH with a low DL of 0.065 fM in whole plasma samples [184]. Humanity has been afflicted by a variety of viral diseases throughout history, from antiquity to the present. Due to the present pandemic’s tremendous losses in terms of lives, money, and necessities, the scientific community has reported various prevention and treatment techniques. Along with a simple synthesis method for GO-Au NC, the fabrication of two immunosensors for the simultaneous detection of SARS-CoV-2 antigen and antibody is also presented. Because of their abundant functional groups for efficient biomolecule binding, high conductivity, and vast surface areas, the synthesized GO-Au NC demonstrated remarkable qualities for sensing applications. Using the sensitive DPV technique, both immunosensors were successfully employed to identify SARS-CoV-2 (Figure 6B) [185]. In redox electrolytes, the dual detection of both immunosensors was conducted using synthetic samples, and the results were confirmed using patient serum and nasopharyngeal swab samples. When compared to the SARS-CoV-2 antibody immunosensor, the obtained DL for the antigen immunosensor was 3.99 ag mL^−1^. These findings demonstrated the great potential of the developed immunosensors for precise, quick, steady, and sensitive determination of SARS-CoV-2 antigen and antibody in clinical POC applications. Early detection and treatment of malignant melanoma depend heavily on the sensitive and precise detection of tumor biomarkers. Highly desirable immunoassay features include an upfront sensing interface and high sensitivity. Recently, the S-100B protein (a tumor marker for malignant melanoma) was sensitively detected using a simple EC immunosensor based on chitosan (CS)-RGO-GCE. To offer a biocompatible, conductive, and simple chemically modifiable matrix for further encapsulation of the antibody. The immunorecognition interface was produced by grafting anti-S-100B antibodies onto the CS molecules using a simple glutaraldehyde cross-linking method. S-100B was determined by a decrease in the current signal from solution phase probes, which was brought on by increased insulation and steric hindrance caused by the formation of antigen–antibody complexes at the electrode interface. The developed immunosensor displayed good stability and high selectivity. Additionally, the sensor could be used to accurately detect the S-100B protein in actual human blood serum samples [186]. Compared with GO the RGO realizes sophisticated conductivity. Ma’s group used the RGO-thionin-hemin-Au NH as labels to develop the voltammetric immunoassay for neuron-specific enolase detection [187]. The three-layered effects of ordered labels on RGO boosted sensitivity: The excellent conductivity of RGO contributed to electron transmission, RGO was used to increase the consistency and EC performance of hemin, which was loaded on the la-bels, and RGO was used to restrain a large number of signal species with its particular surface area. The SWV responses showed an increase in signal with elevated analyte concentration and obtained 0.026 pg mL^−1^ DL. Not only, GO extensively used, but the graphitic C_3_N_4_ NS were executed to enhance signal species profited from its astonishing biocompatibility, atomic thickness, and high surface exposure for coupling of biomolecules signal species [188].

Immunosensors benefit from the sensitivity and selectivity of Ab/antigen interactions to capture and sense the bioanalytes in biotic or ecological samples, yet they frequently include time-consuming measures and require at least two Ab in charge of the whole detection method. Zheng’s group [190] designed a cascade reaction immunosensor for carbohydrate antigen (CA 24-2) detection constructed on a multifunctional zeolitic imidazolate framework (ZIF). The MB-GOx-ZIF-8/Au-RGO was fabricated by a coprecipitation approach for loading signal species and enzymes. The ZIF-8 coupled with tagging antibodies was used as an immunoprobe. The immunoprobe activated the cascade reaction to catalyze the Glu oxidation and H_2_O_2_ production. At once, the produced H_2_O_2_ persuaded the disintegration of PABA/polyvinyl alcohol (PVA) films deposited on the substrate, making the PVA breakage away from the PABA improved substrate. Similarly, an EC immunosensing method using AuNPs/MRGO/NF@GCE and a modest immunochemical assay with biotinylated cortisol Ab and HRP labeled with streptavidin were reported. Furthermore, EC detection of cortisol was carried out using the DPV method. Compared with the ELISA assay, the use of cortisol-modified AuNPs/MRGO electrodes exhibited a great enhancement in sensor sensitivity with not drop in selectivity [191]. Similarly, a facile label-free EC immunosensor based on AuNPs/GR NC was reported for sensitive detection of carcinoembryonic antigen (CEA) by monitoring the variation of charge transference resistance triggered by the definite immunoreactions [192]. A new method for improving GR hydrophilicity by modifying it with pyrene followed by a noncovalent attachment of polyethylene glycol was developed. The prominent part of the fabricated immunosensor was enhancing the Au NPs loading using amine-modified polyethylene glycol to boost the electroanalytical performance. The invented EC immunosensor exhibited exceptional EIS performance for CEA detection with wide LR, stability, and good reproducibility. The immunosensor was employed for CEA determination in human serum samples. Furthermore, a WO_3_-RGO NC-based EC immunosensor was testified for the cardiac troponin-I (cTnI) biomarker detection [193]. A modified anti-cTnI/APTES/WO_3_-RGO/ITO sensor was attained via electrophoretic deposition method, trailed by covalent immobilization cTnI-Ab. The diverse electron allocation kinetics of WO_3_-RGO NC exhibited EC performance compared to bare and other control electrodes. The superior EC aptitude was credited to (i) massive oxygen fractions in WO_3_ that augmented the Ab loading capacity, (ii) robust covalent connection of Ab molecules to the WO_3_-RGO matrix via APTES, which improved the device stability, and (iii) strong synergetic effect amid WO_3_-RGO NCs that has improved the electron transmission kinetics. Recently, EC immunosensors based on Mo_3_Se_4_@RGO/ITO and employing acetaminophen as peroxidase substrate was also reported for SR and thyroid stimulation hormone detection in real samples [177,194]. One of the deadliest viruses, dengue fever, has developed into a recurring public health issue in tropical regions. The demand for rapid and precise diagnostic techniques has grown as dengue incidence increase globally. An effective label-free immunosensor platform was made using functionalized Au, hetero atom-doped GR (AuNPs/NSG), and in situ reductions to detect the presence of dengue virus type E-proteins (DENV-E protein). Au (III) was reduced with L-cysteine as a reducing agent. The reported sensing platform showed that more antibodies were immobilized when L-cystine was added. Unconventionally, the highly crystalline AuNP was grafted onto a 2D GR sheet (see Figure 6C) [189]. Under optimal conditions, the immunosensor had 1.6 pg mL^−1^ detection limit. When tested against their antibodies, the proposed immunosensor successfully distinguishes DENV-E from DENV which is closely related to it. In order to compare the effectiveness of a manufactured immunosensor and an enzyme-linked immunosorbent assay, viral E-protein in patient serum samples was tested. In the future, the AuNPs/NSG immunosensor could also be employed as a possible probe for medical diagnostic purposes. Beyond these, efforts should be made for the practical implementation of these immunosensors with real samples of cancer diseases and other vascular problems. In future, the fabricated immunosensing platforms can be miniaturized to advance POC devices for quick, steady, selective, and inexpensive detection of these bioanalytes. Additional DNA sensors and immunosensors based on GR-NCs reported in the literature are presented in Table 2.

### 3.5. Supplementary Bioanalytes Detection

Small bioanalytes play a major role in regular life functions such as signal transmission, metabolism, and genetic expressions. For instance, Glu provides energy and takes part in the metabolism process of a living body. However, low or high Glu amount will cause hypoglycemia or hyperglycemia. Sensitive and fast determination of such a small bio analyte is indispensable for the initial analysis and cure of these ailments. With the advancement in nanotechnology, various EC sensors-based NMs have been developed for small bioanalytes detection [203,204]. With the addition of 3D spongy GR, the electrocatalytic performance of EC sensors based on Glu oxidase (GOx) was significantly improved. Traditional EC assembly of GO on Pt wire successfully produced an innovative, flowerlike microelectrode made of GR wire. The constructed sensor revealed admirable direct electron transmission and Glu sensing ability with acceptable LR and DL of (1 µM) after GOx was later immobilized on spongy GR by adsorption. The EC signal did not appear to change after one month of storage and consistent 100 scan cycles [205]. A non-enzymatic EC sensor based on (3DGR@Cu NPs) was fabricated to detect Glu in human serum. The effect of various Cu NPs based electrodes was analyzed by EIS, CV, and DPV. The 3DGR@Cu showed distinctly improved EC performance toward Glu oxidation in a basic medium as compared to simple 3DGR foam. The Glu oxidation on the Cu electrode involves multistep reaction in which Glu is catalytically oxidized by Cu(III) species to yield gluconic acid, while Cu(III) returns to Cu(II) as follows: [206]

Cu(III) + Glu → Cu(II) + Gluconolactone(8)

Gluconolactone hydrolyzation → Gluconic acid(9)

As a result, the Cu(III)/Cu(II) redox pair is able to detect Glu using nonenzymatic EC and Cu(III)-aided fast electron. Additionally, the ability to detect Glu was influenced by the concentration of Cu-Cu_2_O and Cu active sites in the GR foam. In the presence of competing bioanalytes, the developed sensors displayed outstanding sensitivity and wide LR.

A crucial nutrient and antioxidant AA, often known as vitamin C. It is crucial to a living metabolic system’s defense against oxidative stress. A lack of AA can cause a number of chronic illnesses, including allergic reactions and liver problems. Therefore, the detection of AA is vital in food engineering, and medical and diagnostic applications. For this purpose, a simple EC sensor based on Ni/RGO precursor was fabricated via a solvothermal approach with a uniform 10 nm size [156]. The fabricated composites were characterized by XRD, TEM, SEM, and voltammetry). The modified sensor exhibited remarkable EC performance toward AA detection. Likewise, 3D NCs based on the self-assembled MoS_2_ nanospheres and PANI coated on RGO were fabricated by a one-step hydrothermal approach, tracked by EC catalytic detection of UA, AA in urine, and blood samples. The synergy effects of the active electrocatalytic aptitude of PANI, EC performance of MoS_2_, and the exceptional charge transfer properties of GR imparted superior electrocatalytic activity for the AA and UA oxidation reactions. The sensor showed 22 µM, 0.3 µM DL with high AA and UA selectivity in biological fluids [207]. 97% of protein tissue comprises L-cysteine (L-Cys) for purification and fortification of ROS cells. Deficiency of L-Cys causes liver damage, skin lesions, fat loss, and slow growth.

In contrast, high L-Cys levels may develop an increased risk of Parkinson’s and Alzheimer’s diseases. However, high oxidation overpotential and weak EC response of L-Cys on the bare electrode surface is still problematic for detecting L-cys. To solve this issue, 3D Mn-La/RGO NCs was synthesized by a simplistic thermal approach. The practical application of the modified electrode was studied for L-Cys detection that expressed remarkable electrocatalytic activity. At optimized parameters, the current density of L-Cys showed a linear relation with concentration (0.5 μM–832.5 μM) by the DPV method. Furthermore, the designed sensor was employed for real-time determination of L-Cys in serum [208]. The central nervous system releases the neurotransmitter epinephrine (EP) in reaction to anxiety, agitation, or hypoglycemia. Li et al. [209] reported an EC sensor for EP based on RGO/MWCNT/TiO_2_/Au nanocluster. The hybrid NCs provided outstanding response and low overpotential of EP owing to the exceedingly active sites of GR and quick electron transmission of CNTs. The DPV technique was applied to evaluate the EC performance of EP electrooxidation with an extensive LR of (1 nM–3 µM) and 0.3 nM DL. The designed sensor was utilized in practice to detect EP in urine samples and had good recovery rates. The MIP method was applied once more to locate cardiac troponin I. Myocardial infarction (heart attack), which can be fatal, is known to happen when the heart’s blood flow slows or ceases. Later, numerous researchers looked into alternate approaches. The cardiac muscle protein serves as a biomarker to detect myocardial infarction impairment. the construction of a platform for layer-by-layer sensing based on glutaraldehyde NCs, CNTs, and GNSs. This ground-breaking substance accelerated electron transmission while supporting intricate protein antigenic protein combinations. An EC sensor based on ssDNA/PANI/GR NCs was selected to diagnose the HIV-1 gene [210]. The ssDNA probe was immobilized on the GCE, and the negatively charged HIV-1 phosphate backbone was attached to the electrode surface via π-π∗ interactions. The crossing among the target HIV-1 and the ssDNA probe formed the dsDNA. The electron transference resistance of the sensor was analyzed by EIS using a [Fe(CN)_6_]^3−/4−^ oxidoreduction couple mediator. At adjusted parameters, the EIS value fluctuation was linearly associated with the log of the HIV genes concentration. The results exposed that the addition of PANI improved the GR dispersion in solvent dimethylformamide and significantly improved the stability and conductivity of the sensor electrode. Fan and colleagues [211] were able to report precise sites to a couple of enzymes (Glu Ox and horseradish peroxidase) on a quadrilateral DNA origami with particular inter-enzyme distances on Au electrodes. The Au-S chemistry was employed to generate a programmable, EC-driven biomimetic system covering both electronic and biochemical components. The analyte passes through the enzyme pair and the final product transfers electrons to the electrode surface at the provided specific potential. A stable state current response that documented the full enzyme activity was interpreted from the flux of enzymatic reactions that depended on distance. Due to its controllability, high-located accuracy, and programmable surface when integrating NPs or bioanalytes, this 2D DNA origami may be used as a pedestal to create new sensing methodologies. The advantages of paper-based electrodes over other electrodes include their affordability and ease of preparation, which makes them appropriate for mass production. They are resistant to environmental conditions and do not oxidize easily. After this thought, an EC sensor based on (GO-C_3_N_4_)/paper and a diagnostic device was developed for DNA determination of norovirus [212]. The fabricated sensor material was evaluated by various techniques including FESEM, XRD, and X-ray. The modified electrode with probe DNA was investigated for EC performance via CV and DPV. Under optimized scan rate, temperature, and response time the modified electrode showed high specificity in the existence of unrelated sequences, wide LR, and the lowest DL of 100 fM toward norovirus detection. The fabricated sensor was inexpensive, highly specific, easy to make, and required an analysis time of 5 s only. Similarly, an EC sensor was reported for Hepatitis B virus detection based on nanoflowers of Cu_3_(PO_4_)_2_-BSA-GO NCs. The multifaceted biosensor layers offered enough binding points and an adaptive context for signal intensification and biocompatibility. The EC DNA sensor displayed a DL of 1100 copies mL^−1^ [213]. Mills and colleagues developed an exclusive podium to expose two different DNA sequences (Zika and Dengue) via a single EC sensor [214]. The sensor was made up of two DNA-specific adaptor components and a generic stem-loop probe attached to an Au electrode. These adaptor strands delivered strong binding affinity and precise recognition after hybridizing to one nucleic acid. The universal probe was quickly redeveloped using a water and urea solution. With just one EC sensor, the newly designed sensor provided a sensing platform for the quick and economical detection of any RNA or DNA sequence.

## 4. Conclusions and Perspective

In summary, we have debated the recent advancements in the developments of EC sensors based on GR for the detection of cancer biomarkers, neurotransmitters, DNA, proteins, and various other bioanalytes used as promising biomarkers in disease diagnostics. These EC sensors are often used to detect bioanalytes in various actual samples such as serum, saliva, blood, tears, and live cells. The working principle of these EC sensors involved the interaction of GR-based NCs with various metal oxides, conducting polymers, proteins, DNA, antigen/Ab coupling by employing different voltametric, impedance, and AM techniques. At this point, we critically discuss the EC detection mechanism of significant cancer biomarkers (H_2_O_2_, NO, H_2_S) and neurotransmitters (DA, Ach, ST), DNA sensors and immunosensors in detail with a special focus on recently developed state-of-the-art devices. For increasing the sensitivity of EC DNA and immunosensors, a cogent technique of signal intensification mode and elastic selection of functionalized NMs is extremely promising. The perks of operating range, morphology, and biocompatibility make GR-based NCs suitable for sensitivity improvement methods. In particular, making labels with an increased loading capacity of signal molecules and employing cascade or catalytic reactions activated by these labels are two extensively used approaches. These high-tech sensing podiums could pave the way for immediate, precise, specific, complex, and onsite detection organized with various diagnostic tools for cancer, brain, cardiovascular, and other unparalleled deadly diseases. The biomarker-based analysis potential of EC sensors will be the appealing method in amalgamation with medical annotations and risk aspects to treat the affected role conferring on the sternness of their sickness. Of late, EC biosensors have effectively detected various promising disease biomarkers from biotic matrices. Thus, EC sensing approaches are the finest way to identify the disease at its initial phase.

The study of GR-based NMs is a topical research interest that offers considerable potential in electroanalytical chemistry. However, to smooth out the practical applications of GR-based NMs and enhance their EC sensing capabilities in the future, there is still much work to be done. A number of current challenges, including more straightforward synthesis, meticulous processing, functionalization, and thorough characterization of GR assembly at the nanoscale, should be given careful consideration. Additional research is needed to better understand the electrical characteristics, the effect of defect formation on GR conductivity, the role of electron transport at the GR/electrode interface, and the development of GR-based POC devices. Similar to the above, simulation techniques are advantageous and helpful for creating the optimal substrate for commercial requirements. Molecular simulation can be used to forecast the interaction and structural changes brought on by contact with biomolecules, medicines, nucleotides, etc. Small changes in the GR assembly could not be constituted in wet investigational circumstances, despite the fact that the same collaboration may be demonstrated using simulation techniques. These traits might be able to predict structural faults as well as interactions between neighboring molecules. These modifications could be useful in constructing a systematically sound system that most likely identifies interactions or makes recommendations on the specificity of GR and medicines or biomolecules. The diverse surface features of GR-based materials, which have been shown to alter the high performance of EC processes, may aid in the development of ultrasensitive biosensors. We will create extremely selective, sensitive, and repeatable sensors by altering and decreasing GO and manipulating its electrical characteristics. We believe that advancements in nanotechnology, electrochemistry, natural science, and POC devices will aid in the growth of the next generation of extremely accurate, sensitive, and dependable EC sensors. As a result of the enormous attention paid to physical and psychological well-being, innovative EC DNA sensors, biosensors, and immunochemical assays for clinical analysis are gaining prominence. Furthermore, reagent-free biosensors, single bioanalyte detection, and miniaturized detection devices are important goals for improved health and quality of life. Furthermore, the use of portable GR-based EC sensors in POC testing can aid in medical care and environmental remediation, as well as enable quick, sensitive, and precise on-the-spot analysis.

## Figures and Tables

**Table 1:** EC sensors based on GR NCs for various bioanalytes detection.

Bioanalyte	Electrode Materials	Technique	Sensitivity (μA μM^−1^)	DL (nM)	Reference
H_2_O_2_	GR/Ag NPs	AM	15.5	147	[86]
H_2_O_2_	MnO_2_@GR NSs	AM	422	100	[131]
H_2_O_2_	NiCo_2_S_4_@RGO	AM	118.5	109	[132]
H_2_O_2_	Pt/ZnFe_2_O_4_/RGO	AM	−	100	[133]
H_2_O_2_	AuNPs/GR/CNT	AM	−	17	[134]
H_2_O_2_	NiN_3_@GR aerogel	AM	905.6	40	[135]
H_2_S	CuS/RGO	AM	−	300	[136]
H_2_S	RGO/Fe_3_O_4_/Cu_2_O	DPV	−	0.002	[137]
NO	RGO/CeO_2_	AM	1676	9.6	[138]
NO	APBA/RGO	AM	37.6	5	[99]
NO	Au NPs/ERGO	AM	5.38	133	[139]
DA	Pristine GR	CV	10.3	200	[140]
DA	GR	DPV	0.7	144	[141]
DA	Zn-NiAl LDH/RGO	DPV	−	0.1	[142]
DA	GR/GQDs	DPV	14.25	30	[143]
ST	3D-RGO/Fe_3_O_4_/HP-β-CD	DPV	−	3.3	[144]
ST	GR-PEDOT-Ag	DPV	−	0.1	[145]
ST	GQDs@2D hBN	DPV	−	0. 02	[146]
Ach	Fe_2_O_3_/PEDOT/RGO	AM	0.39	4	[126]
Glu	Pristine GR/GOx	AM	16.3	800	[147]
Glu	Cu_2_O NPs@3DGR	AM	1.8	10	[148]
Glu	Ag–PANI/RGO	AM	−	70	[149]
UA	GR NSs	DPV	−	3	[150]
UA	N doped RGO	DPV	−	200	[151]
UA	MIP/RGO NCs	DPV	−	3.2	[152]
AA	N doped GR/Ag/PANI	AM	−	800	[153]
AA	AuNPs/RGO/SPCEs	AM	−	1700	[154]
AA	MoS_2_/MWCNTs/ZnO/GCE	AM	−	1000	[155]
AA	Ni/RGO/GCE	AM	−	600	[156]
L-Cys	NiO NPs/N-RGO	AM	0.051	10	[157]
L-Cys	CuFe_2_O_4_/RGO-Au	CV	79.23	59	[158]

**Table 2:** List of various DNA sensors and immunosensors based on GR-NCs reported in the literature.

Bioanalyte	Working Electrode	Transduction Technique	LR	DL	Reference
DNA	MnTPP/RGO/GCE	EIS	100 aM–10 pM	6 pM	[195]
DNA bases	Cu/NRGO/GCE	DPV	0.132–1575 µM	-	[196]
DNA	GR-fc-ssDNA	EIS	−	10 mol L^−1^	[197]
DNA	CRISPR/Cas9/3DGR/AuPtPd	DPV, EIS	0.01–500 pM	0.13 pM	[163]
P-53 Protein	Thiolated-GO-streptavidin-AuNPs-GCE	DPV	0.2–2 pM	30 fM	[183]
cTnI	GO–Ph–AuNP/Ab	AM	0.05–3.0 ng mL^−1^	0.05 ng mL^−1^	[198]
cTnI	anti-cTnI/PRGO/GCE	EIS	0.1–10 ng mL^−1^	0.07 ng mL^−1^	[199]
CA-42	MB-GOx-ZIF-8/Au-RGO	SWV	0.001–1000 U mL^−1^	69.3 U mL^−1^	[200]
Bilirubin	SPCE/MWCNT/GR	DPV	0.1–600 µM	0.018 nM	[201]
Mayoglobin	Ab/GQD/SPE	EIS	0.01–100 ng mL^−1^	0.01 ng mL^−1^	[184]
CA-19-9	Au@Pd-GR/Thi-Ab2/HRP	DPV	0.015–150 U mL^−1^	0.06 U mL^−1^	[202]

## Data Availability

Not applicable.
